# Burying power: New insights into incipient leadership in the Late Pre-Pottery Neolithic from an outstanding burial at Baʻja, southern Jordan

**DOI:** 10.1371/journal.pone.0221171

**Published:** 2019-08-28

**Authors:** Marion Benz, Julia Gresky, Denis Štefanisko, Hala Alarashi, Corina Knipper, Christoph Purschwitz, Joachim Bauer, Hans Georg K. Gebel

**Affiliations:** 1 Institute of Near Eastern Archaeology, Free University, Berlin, Germany; 2 German Archaeological Institute, Berlin, Germany; 3 Department of Archaeology and Museology, Masaryk University, Brno, Czech Republic; 4 Cultures et Environnement, Préhistoire, Antiquité, Moyen Âge (CEPAM), Université Côte d'Azur, Nice, France; 5 Curt-Engelhorn-Centre Archaeometry gGmbH, Mannheim, Germany; 6 Albert-Ludwigs-University, Freiburg, Germany; University at Buffalo - The State University of New York, UNITED STATES

## Abstract

In 2016, an extraordinary burial of a young adult individual was discovered at the Late Pre-Pottery Neolithic B (LPPNB, 7,500–6,900 BCE) settlement of Baʻja in southern Jordan. This burial has exceptional grave goods and an elaborate grave construction. It suggests discussing anew reconstructions of early Neolithic social structures. In this article, we will summarize former theories on the emergence of leadership and hierarchies and present a multivariate model according to which anthropological and archaeological data of the burial will be analyzed. In conclusion, we surmise that early Neolithic hierarchization in southern Jordan was based on corporate pathways to power rather than self-interested aggrandizers. However, some aspects of the burial point to regional exchange networks of prestige goods, a trait considered characteristic of network based leadership. In line with anthropological and sociological research, we argue that pathways to power should be considered as relational processes that can be understood only when comparing traits of the outstanding person to her/his social environment.

## Introduction

In 2016, a very elaborate single adult burial was discovered at the Late Pre-Pottery Neolithic B (LPPNB) site of Baʻja (30°24'48''N, 35°27'40''E) in southern Jordan (S1 Fig). Its outstanding characteristics, such as the elaborate grave construction and the exceptional grave goods, initiated a reconsideration of the emergence of early Neolithic hierarchization and leadership. The reconstruction of the complex burial ritual gives important clues for an improved understanding of the relational character of early Neolithic leadership. Pathways to power are a recurring topic in anthropological and archaeological research [1–7]. With few exceptions (*e*.*g*. [8–10]), many anthropologists still assign physical and coercive power as one of the main incentives to hierarchical organization with (aggressive) aggrandizers being considered the motor of social evolution [6, 11–12]. However, empirical support for the proposed universal link between offensive warfare and the emergence of states is still lacking [13–14]. There is increasing evidence in socio-political research that political leadership among small-scale traditional horticulturalists, but also in larger complex societies is achieved by enhancing cooperation with non-kin, by coordination of collective action, representation of group interests, as well as by successful conflict mediation [7, 14–18].

According to socio-anthropological research, two types of leaders thus seem to be at opposite ends of the scale (Table 1): leaders who gain power by informal modes of acclamation and others who conjure fear and apply coercion. The latter expect unlimited loyalty and legitimize their power by the “need” of their group for a “strong hand” to protect and guide them; in general, these types of leaders promote specific power-supporting confined group identities with clear-cut distinctions of one’s own group and “strangers”. Accumulation of wealth or its inequitable redistribution by the leading group may augment economic disparities between the leading group and the community. Ideocratic structures may promote these types of leadership more than communities in which relational social structures and habitus determine personal and social identities [19].

**Table 1:** Characteristics of two opposed idealized types of leadership, their community members and their social organization.

	*Primus inter Pares*	Aggrandizer
**Characteristics of the behavior of the leading agencies**^**a**^	Displays technical, medical, art-related or diplomatic competences	Conjures fear, perhaps a good warrior or strategist
	Leadership by charisma, acceptance or approval	Applies control and coercion
	Highly skilled mediator and manager of conflicts	No specific mediating competences other than displaying a “strong hand”
	Socializer, producer of new contacts with outside groups	Elitist contacts to interregional peers and distance from subordinates
	Allows individualism, variety and personal freedom	Requests unlimited power and loyalty
	Good in building alliances	Belligerent, seeks outside conflicts
	No need of “symbols of power”	High need of “symbols of power”
	Rather equal redistribution	Accumulation; uneven redistribution
**Matching characteristics of community members**	Members of the community possess specific competences (e.g., craft), replacing them is difficult	Subordinates possess few skills, no essential competences, they can be replaced easily
	Open access to knowledge and resources	Restricted access to knowledge and resources
	Developed self-assurance	No self-assurance
	Self-motivated	Motivated by fear or coercion
	High intrinsic loyalty and identification with the community	Superficially imposed loyalty and extrinsic identification with the community
	No need of strong ideology (“ideology”is intrinsic)	Need for strong ideology to keep subordinates in line
**Social organization**	Promotes heterarchical structures	Promotes command structures, promotes “cone-shaped”structures

These two types are idealized poles of a continuum of various relationships between leading agencies, members of the community and socio-political organization. No community will ever match with these idealizations, but each can represent a specific mixture of varying features of both types.

^a^ This table has been conceived by JB.

At the other end, there are leaders receiving and maintaining their power by non-enforced, but self-motivated optional acclamation from their peers for being good mediators able to steer peer pressures and coordinate interests as *primi inter pares* (as is characteristic for *e*.*g*. habitus societies *sensu* Gebel [19]). *Prototypicality* of the leader–the leader is perceptually assimilated to the group–and *social attraction* are said to be main factors in successful leadership ([20]; see also [21]). Although such groups are generally more heterogeneous than groups based on coercive power, social processes of imitation and popularization can lead to a rather homogeneous appearance but without strict canonization. The more equitable redistribution within this type of societies may lead to a more homogenous distribution of economic wealth. Moreover, borders tend to be less marked. Leadership by acclamation of the habitus-type (see above) requires more social negotiation and less ideocratic input [19]. Leadership can be “distributed across multiple group members or concentrated in a single individual” ([21], see also *Definitions*).

A rather similar distinction was made earlier in anthropological research. Following Renfrew’s [1] differentiation of group-oriented and individual leaders, Feinman ([22]; see also [23]) elaborated “two ends of a continuous range of pathways to power and inequality”: corporate and exclusionary strategies. Group-oriented (corporate) leaders gain their power via the support of their group members; power is less overtly demonstrated but instead shared in several social entities. In contrast, self-interested individuals (exclusionary strategies) gain their power through the support of their equals, from familial ties or other groups via a network of other aggrandizers. They rule autocratically and the “economic foundations of power tend to have their roots in the spoils of war, long-distance exchanges, or other forms of easily concentrated wealth.” These two different pathways to power affect how identities of leaders are conceived and publically manifested and possibly also on how group identities are represented by symbolic media: from architecture to rituals and imagery.

It goes without saying that such a division in two opposed types of leadership is a heuristic means and a strong simplification. Every community comprises many diverging agents of power such as informal and structural power, healing, magic, ritual and economic power. These institutions, groups or individuals may, but do not have to, converge with leadership.

The personal traits of the leader thus represent only one aspect of the social structures of leadership. To understand the character of leadership, it is necessary to consider his/her relation to the community. Socio-neurobiological research and evolutionary anthropology have shown that accessing, establishing and maintaining power is always a relational process [20, 24]. Leadership depends on the prevailing discourse and ethical concepts. Even if new leaders deliberately represent themselves in clear contrast to traditional concepts, they are still children of their times (for a summary on social constructionism see [25]; for the Neolithic Gebel [26] used the term *milieu*). This implies that pathways to power are not solely based on the agency, abilities and skills of power-seeking aggrandizers (*cf*. [6, 11]), but also on the group and its socio-economic, ideological and natural environments (e.g. [18]). Even in non-autocratic societies, when conflict and fear of other groups increases, social processes can lead to the promotion of charismatic and even autocratic self-interested leaders [20, 22, 27]. These relational aspects of leadership have considerable consequences for the studies about pathways to power.

To gain insights and to understand the great variety of the processes of how human communities evolved from rather egalitarian systems to stratified societies, a diachronic approach is necessary and has to comprise (pre-) historic data of various case studies. Archaeological research about early farming communities can provide invaluable insights on how power was established at the transition to sedentism and production–one of the turning points of human history.

In the prehistoric archaeology of the Near East, social organizations of early farming communities have long generated a controversial discussion (e.g. [3–4, 19, 28–32]). On the one hand, it has been argued that a “centralized and powerful decision-making authority or apparatus” would have been necessary to manage increasing population densities and organize living in permanent large-scale settlements [32]. Social alienation due to increasing population densities [33] made the mitigation of conflicts a crucial social requirement for the successful transition to village farming communities [26, 34–37]. Exchange of commodities might have contributed to the mitigation of emerging conflicts in productive milieus [26]. The possibility to accumulate, store, display, retain or distribute goods probably enhanced the potential to manifest and establish social differentiation by “objectification” [16–17, 23, 38]. Many archaeologists and anthropologists therefore still consider the beginnings of agriculture and the production of surplus or differential access to fertile land as the main driver of hierarchization ([4, 17, 23, 29, 39], *cf*. [11, 18, 40]).

Early Neolithic communities in the Mediterranean zone have been reconstructed as “territorially organized, non-egalitarian […] ‘tribes’” ([41], see also [3]) whereas groups of the arid zones were considered “egalitarian”. Burial rituals of the LPPNB suggested the veneration of specific persons, groups or sodalities [42–44]. Skull deformation might even indicate that a specific status was ascribed to some individuals at a very young age [45–46]. Imagery and communal architecture from Northern Mesopotamia have been interpreted as evidence for chiefdom [30] or the dominance of clerical elite [47]. In a similar vein, for the developments during the LPPNB at ‘Ain Ghazal, Rollefson [4] suggested “a social hierarchy based on religious control” (p. 150).

On the other hand, architectural homogeneity in the Levant and Central Anatolia as well as some burial practices have been interpreted as evidence for rather egalitarian structures that level emerging social differentiation [48–50]. Gebel [19] pointed out that the Neolithic relational communities of the southern Levant tended to promote flat hierarchies, in which leading community members cooperated in the decision making processes for the community. This is in contrast to more ideocratic groups of the Northern Levant, which needed institutions to control doctrinal ethical or ideological concepts. These differences in interpretation are only partly due to different perspectives of research. They support the idea that there were multiple pathways to socio-political leadership driven by differences in environment, social conditions, mode of procurement and production as well as access to knowledge.

Considering these relational aspects of leadership and the obviously different pathways to power, our aim is to present the empirical evidence for the special burial from the LPPNB site of Baʻja, in southern Jordan, in relation to the group and regional traditions. Within this theoretical model, the extraordinary single burial will be analyzed according to a catalogue of criteria (S1 Text). In light of these new empirical data, we will suggest a more detailed hypothesis on how hierarchical systems may have developed during the LPPNB in the southern Levant. Our conclusions should not be considered as universal, but rather contributing an example to the variety of incipient hierarchization [17, 28, 47, 50–51]. Our study aims to provide a systematic and holistic analysis as well as an understanding of emerging social differentiation.

## Definitions

For this case study from the LPPNB, we prefer a neutral term–‘outstanding person’–instead of the terms ‘powerful authority’ or ‘leader’ since we cannot specify yet the kind of power or status with which the person was endowed. Extraordinary burials should not be interpreted as “simple proxies for social structure” [18]. As we do not have any imagery or anthropological records that allow us to reconstruct the role of the person, it is not possible to judge from the archaeological data her specific power or function in society. What is observable about an outstanding person are anthropological specificities or a special status indicated by material wealth or specific objects. For the Neolithic, it seems anachronistic to differentiate between social, political or spiritual leadership, despite the fact that these differentiations might have been emerging during the early Neolithic. In addition, the archaeological records do not yet allow a clear differentiation between these three analytical categories.

*Prestige* is understood here as achieved eminence due to extraordinary characteristics, skills, possessions or behavior of the individual. *Social status* implies a social rank or role regulated by conventions, duties and privileges of a social entity. In contrast to prestige, status can be inherited or achieved. Prestige often correlates with status but does not have to. People can hold a certain status without having prestige and *vice versa*.

*Leaders* are defined as “individuals who are accorded differential influence within a group over the establishments of goals, logistics of coordination, monitoring of effort, and reward and punishment. Leadership can be distributed across multiple group members or concentrated in a single individual” (p. 539) [21]. Access to power can range from *prima vista* covert influence to active coercion.

Following Willer *et al*. [52], we define chiefdom as a “kin-based ranked polity” (p. 427) with an institutionalized supra-local leading authority, irrespective of how power is achieved. This is in strong contrast to the definition by Carneiro [6] who considers chiefdoms as “first and foremost a political and military creation, a form of polity brought into being and held together with an iron hand by a successful war leader” (p. 26–27). Sharing the concerns of Morton Fried, we avoid the term “tribe” as descriptive for the structural characterization of a community since it refers rather to the kind of familial relationships within a community [4] than to characterize a socio-political entity. We therefore prefer to speak of segmentary communities for flat-hierarchical large communities to indicate that the segments of these societies are relatively equal, irrespective of whether the segments are clans, lineages or moieties or other social entities. Within these entities, ranking is often determined by age, sex, kinship or provenance.

We use *doctrinal* here as related to indoctrination of canonized forms of imagery, communication or belief systems [19]. Whereas some Neolithic communities of the Northern Levant seemed to support a doctrinal mode of communication and incipient ideocratic structures (*sensu* Gebel [53]), confined habitus communities of the Neolithic were characterized by repetitive daily practice that may have built, over time, strong traditions, almost identical to doctrinal media but based on imitation and transmission of corporate belief systems and habitus. This should not be confused with the differentiation of an imagistic and doctrinal mode of religion established by Harvey Whitehouse [54]. For the description of the ethos of Early Holocene communities from the Northern Levant, we prefer to use the more neutral term *ideocratic* [19], meaning the ruling of generally accepted and unreflected ideas promoting social coherence between groups not necessarily knowing each other [37]. This is different from Bourdieu’s term *doxa* that implies a societal ethos promoted and ruled by institutionalized authorities, such as priests, kings, military rulers, *etc*.

## Empirical data

**Fig 1:**
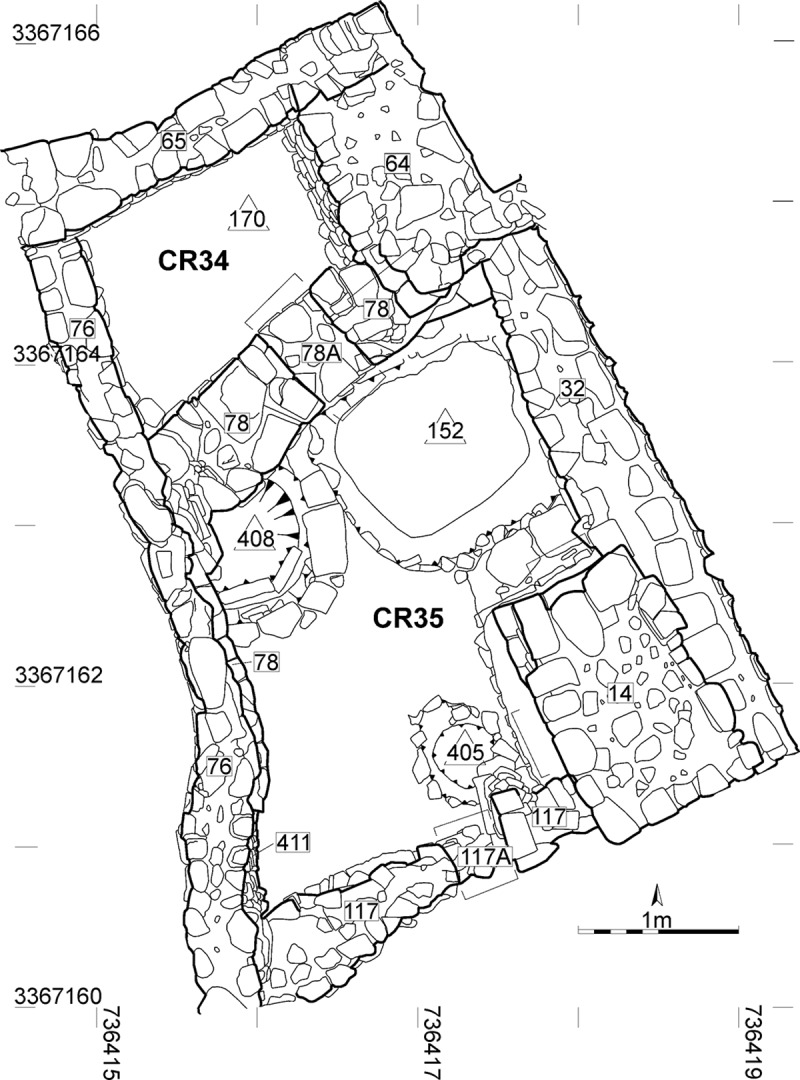
Position of the graves (Loci C10:408/405/152) within Room CR 35, at Baʻja. Printed under a CC BY license, with permission from ex oriente e.V., with permission from Boris Borowski, Katie Tucker and Christoph Purschwitz, 2016.

**Fig 2:**
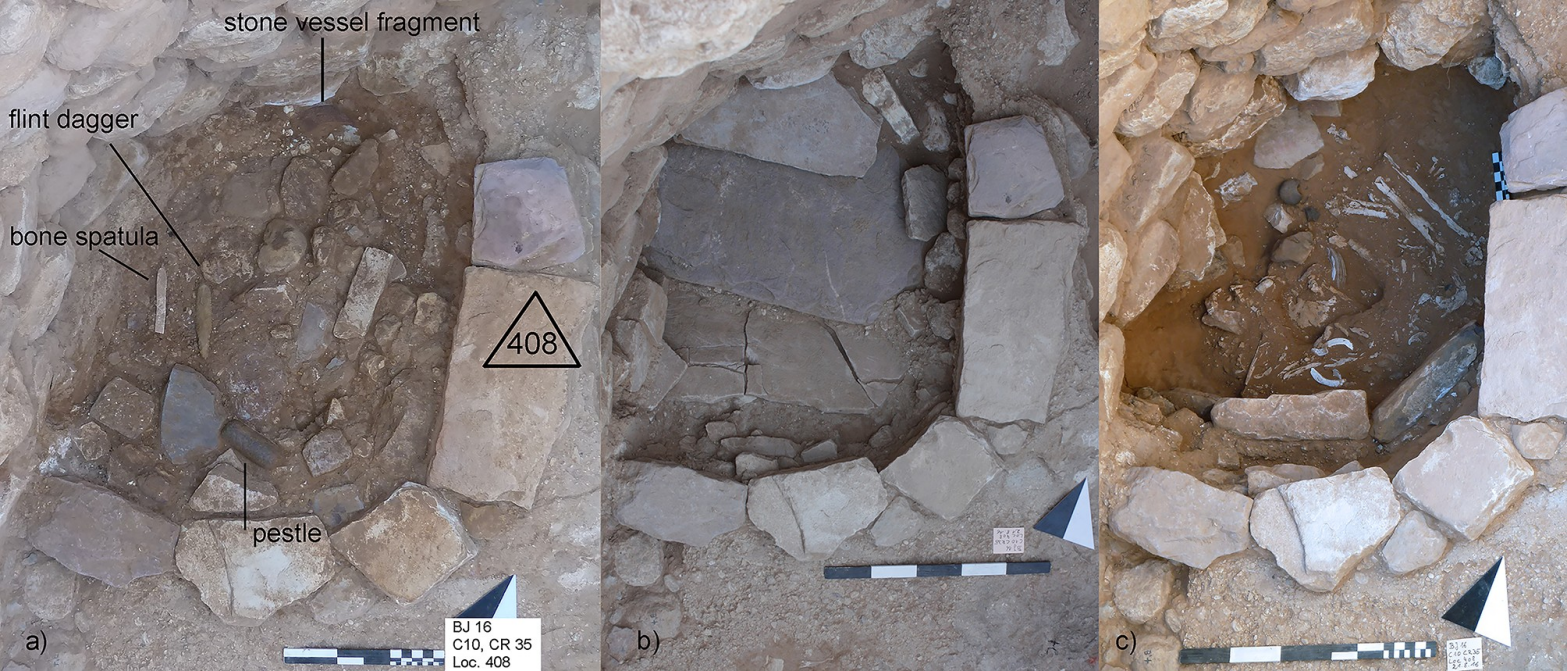
The burial of Loc. C10:408. a) with the embedded objects in the upper grave cover. Republished from ex oriente e.V. under a CC BY license, with permission from Hans Georg K. Gebel, 2017; b) with the stone slab cover, c) after uncovering the skeleton with its grave goods. (Photos: HGKG, MB).

The Baʻja Project was directed from 1997–2016 by H.G.K. Gebel (HGKG) and it has continued since 2018 under the co-directorship of HGKG, M. Benz (MB) and C. Purschwitz (CP) [55]. The outstanding Burial Loc. C10:408 was discovered in Room CR 35, Area C [56] (Figs 1 and 2). Despite structural similarities in grave construction to the collective burials of the site, it is the first primary single burial discovered in a clear settlement context at Baʻja [57–59].

### Construction details of the grave and reconstruction of the ritual process

The construction of the grave and the funeral ritual will be described in chronological order of events (Table 2). The place of the burial was chosen in the north-western corner of the room in which two other burials existed or were later added (Event 1; [56]). Although the chronological order of the different burials remains unclear, they belong to approximately the same use phase. Loc. C10:408 is also close to Loc. C10:170, a collective burial in the adjacent northern room of the building. At first, a 72 cm (N-S) large and 60 cm wide (E-W) and about 50 cm deep burial pit was cut through the terrazzo-like floor Loc. C10:403 and its sub-construction of large pebbles (Loc. C10:410) into the hard playa-like natural sediment (Event 2). The pit partly undercut the wall (Loc. C10:78). The southern border of the pit was fixed with two vertical stone slabs whereas on the northern border a slight step had been dug into the sediment in order to place the grave cover on top. A low half-circular wall of stones was built confining the southern and eastern part of the grave. Probably during the burying ritual, a fire was lit on the floor (Loc. C10:403) adjacent to the burial (Event 3). The ash layer and traces of this fire could be clearly seen during the excavation between the floor and the grave’s final sealing (Fig 2B), but it decayed immediately after excavation. The sealing of this ash layer must have taken place immediately after the fire expired. After the corpse had been laid into the pit with its personal belongings, a macehead had been placed next to his shoulder and deliberately smashed, so that it was broken in two halves and several small chips (Event 4) (Fig 2C). The grave pit was covered with three large overlapping red and white Ordovician sandstone slabs (Event 5, Fig 2B). Traces of charcoal were observed on the northern slab indicating the use of fire during the burial ritual (Event 6, see also Event 3). In a next step, large stones embedded in reddish-brown sand were used to cover the slabs. Within this layer, several objects were placed (Event 7) (Fig 2A). At the end, the final sealing was added with a layer of reused flint- and limestone small-sized gravel that also partly covered the stone border of the grave and the ash layer (Event 8; see also Event 3). The gravel layer ran onto Walls Loc. C10:411 (in the south) and Loc. C10:78 (in the north). This observation proves that the burial was younger than both walls. On top of the gravel layer, a patch of coarse-grained dark-grey sand was deposited in the eastern part of the grave (Event 9). Finally, white plaster was applied, probably on the whole grave, even though it was preserved only in some parts (Event 10). Radiocarbon data confirm the use of this space as a burial area at the end of the LPPNB, between 7071–6684 cal BCE (95.4%) (Table 3). A possible old wood effect of the sample MAMS 30314 due to juniper charcoal cannot be ruled out. However, the date overlaps considerably with the date of a small twig (MAMS 30315) from the nearby double child burial Loc. C10:405, wherefore the probability is rather low. Both graves were dug into the same plaster floor but their precise stratigraphic relation remains unclear (Fig 1). This uncertainty and the plateau of the calibration curve obstructed further statistical operations (S3 Fig).

**Table 2:** Reconstructed sequence of events for the burial ritual of Loc. C10:408, CR35 at Ba'ja.

Events^a^	Activities
**1**	Choosing the burial area in the north-western corner of the basement of Room CR 35. The space was probably transformed into a burial area, given that a double child burial (Loc. C10:405) and a collective burial (Loc. C10:152) were placed in the basement of this architectural entity too;
**2**	digging the pit through the terrazzo-like floor (Loc. C10:403) into natural sediment, partly below Wall Loc. C10:78; setting two vertical stone slabs on the southern border of the pit and aligning the pit’s border with a half circle of stone slabs;
**3**	lighting a fire outside the grave (if the fire does not relate to the burials of Loci C10:152, C10:405 or C10:170 next to burial Loc. C10:408 and in the adjacent room to the north);
**4**	burying the dead with its personal outfit, destroying the macehead;
**5**	covering the pit with three stone slabs;
**6**	use of fire or of charcoal on the stone slab (MAMS 30314: 8039 ±27 BP see Table 3);
**7**	covering the slabs with large stones and deposition of objects (flint dagger, arrowheads, pestle, stone bowl fragment, bone spatula);
**8**	covering with re-used terrazzo-like floor material, filling the gaps in the wall above the stone slabs
**9**	covering with dark-grey sand inside the stone slab half-circle;
**10**	adding white plaster on the southern edge (possibly originally extending over the whole grave–but not preserved);
**11**	decay of body with the head falling on the chest, turning the mandible upside down and possibly other slight taphonomic movements;
**12**	intrusion of fine sand and fine gravel and collapse of the stone slabs.

^a^ modified after Gebel et al. [56].

**Table 3:** Radiocarbon data from burial Loc. C10:408 and the double infant burial Loc. C10:405.

Lab ID MAMS	Context Ba‘ja C10, CR35	^14^C age BP ±1σ	δ ^13^C AMS [‰]	cal BCE 95.4% probability	C [%]	Material	Species
30312	Loc. C10:405 Ind II	**No collagen**				bone unburnt	
30313	Loc. C10:408	**No collagen**				bone unburnt	
30314	Loc. C10:408 BP 97415	8039 ±27	-22.7±0.5	**7071**-6982(54.3%), 6974 6911(18.6%), 6885 **6830** (22.5%)	51.2	Charcoal	*Juniperus*
30315	Loc. C10:405 BP 97422	7928 ±29	-35.1±0.5	**7028-**6931(19.5%) 6920-6877(11.5%) 6860-**6684**(64.5%)	44.9	Charcoal, twig	*Juniperus*

Conventional ages (BP) were calibrated with Oxcal v. 4.3.2. [60], IntCal 13 [61] (S3 Fig), δ ^13^C values were measured in the accelerator and should not be compared directly with radiocarbon data.

The completed grave construction formed a slight elevation of about 10–15 cm from the original floor (Loc. C10:403). Considering the collapse of the stone slab cover, it can be suggested that this elevation was even higher before the collapse. During decay (Events 11–12) the grave pit’s void was filled with fine-grained sand. The taphonomic processes of the corpse indicate a void, at least for a certain amount of time. Traces of gnawing from small rodents might corroborate this observation (see below). Under the pressure of the sediment and the middle stone plate, the southernmost stone slab also collapsed. This must have happened after the sand had infiltrated since the slab did not fall into the grave pit completely.

### The individual

**Fig 3:**
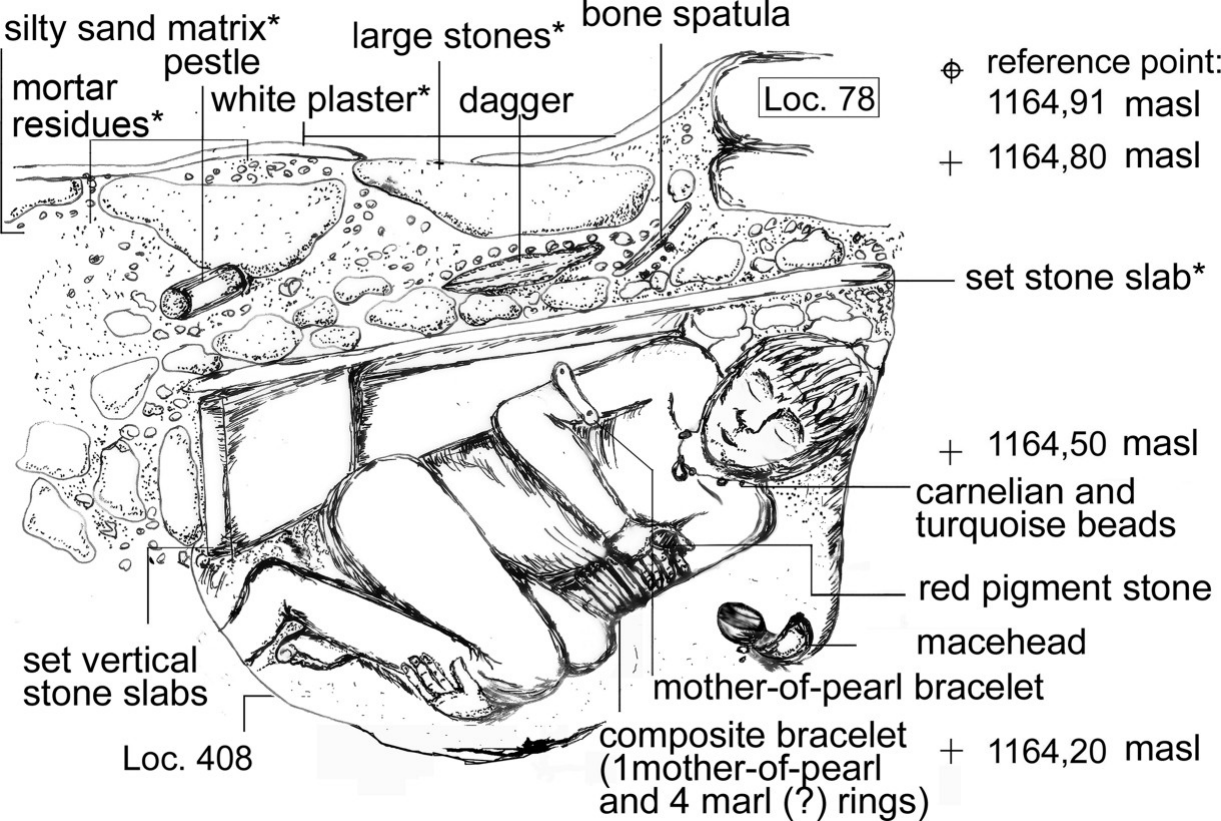
Reconstructed virtual E-W-cut through the burial Loc. C10:408, facing south. * Embedded in the grave cover, printed under a CC BY license, with permission of Marion Benz, 2018.

**Fig 4:**
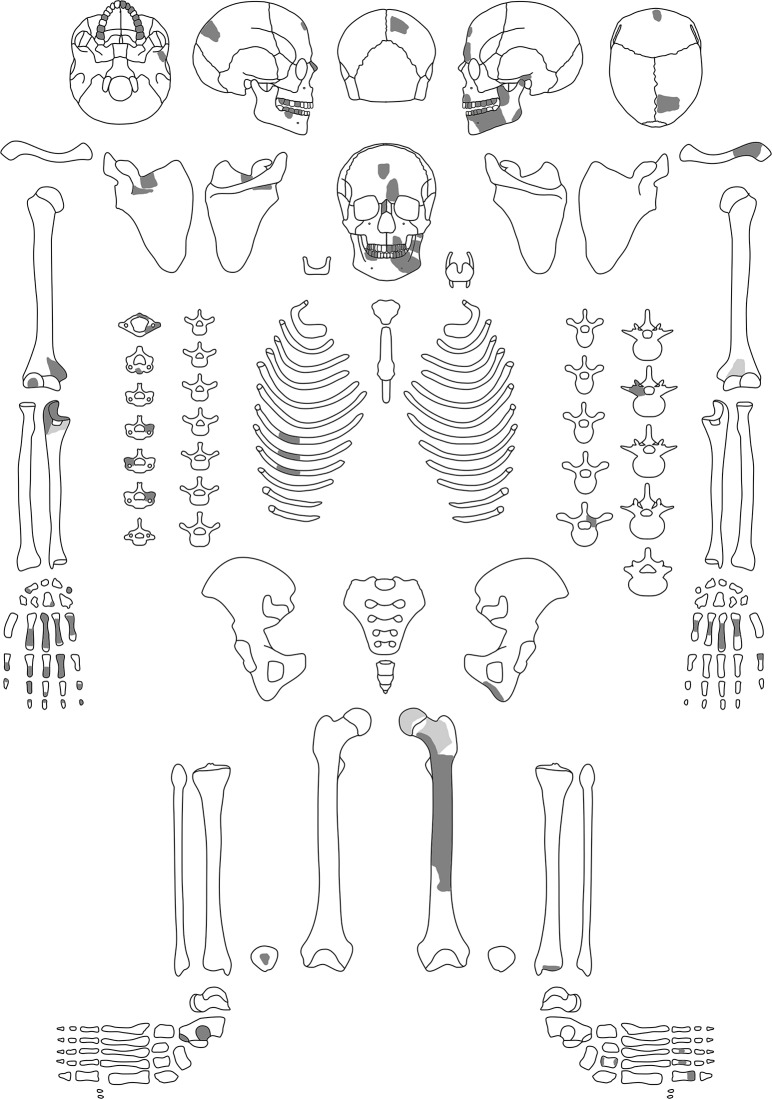
Preserved bones of the individual of Loc. C10:408. Dark grey: well preserved; light grey: poorly preserved elements; printed under a CC BY license, with permission from Julia Gresky, 2018.

The individual was rather squeezed in the pit with its legs flexed (left femur 60°, right 90°) (Fig 3). The orientation was southwest-northeast. Both legs were turned to the left side. The right *tibia* was so close to the right *femur* that some kind of binding seems possible. The left arm was stretched below the legs with the left hand touching the eastern border of the grave pit. The right upper arm lay along the right rib cage and the right lower arm was flexed over the body with the right hand gripping the left upper arm. The torso was lying on its back and the skull had collapsed onto the chest, whereby the mandible had turned upside down. This twisted position of legs, arms and torso is hardly possible *in vivo*. It cannot be excluded that the legs fell to the left side after some ligaments had dissolved suggesting originally a sitting position with the back leaning on the western border of the grave pit. However, the anatomically correct alignments of most bones–except for the head—, do not suggest much taphonomic movements. The skeleton is poorly preserved (Fig 4), the bones extremely friable and their surfaces heavily eroded. All skeletal remains are deposited under the specimen number C10:408 in the Department of Anthropology of the German Archaeological Institute and are accessible to anybody upon request.

There are no clear markers for sex determination preserved except for a prominent *glabella* (score 3–4) [62], relatively big teeth and a strongly built mandible. Taken together these criteria give a slight evidence for a male individual.

For age estimation, dental wear [63] was considered although the environment of the sandy plateau and surroundings suggests more severe attrition due to abrasive elements within the diet. Dental wear of the molars points to an age of 25–35 years. The state of the cancellous bone within the femoral head and neck is dense, which also suggests a young adult age [64].

**Fig 5:**
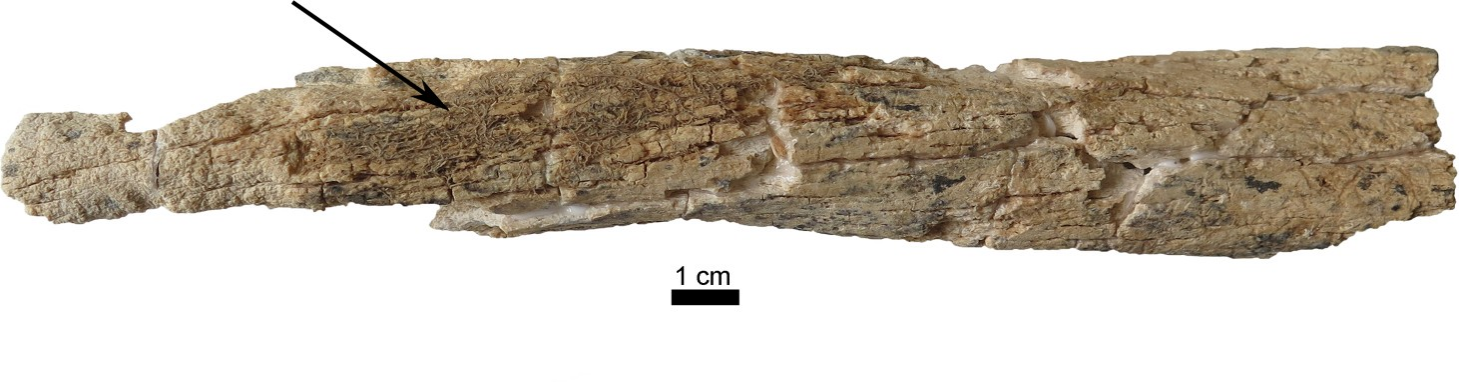
Anterior surface of the left femur of the individual of Loc. C10:408. The femur is showing severe erosion of the surface and cracking of the bone as well as a net of roots closely attached to the surface (arrow). (Photo: JG).

**Fig 6:**
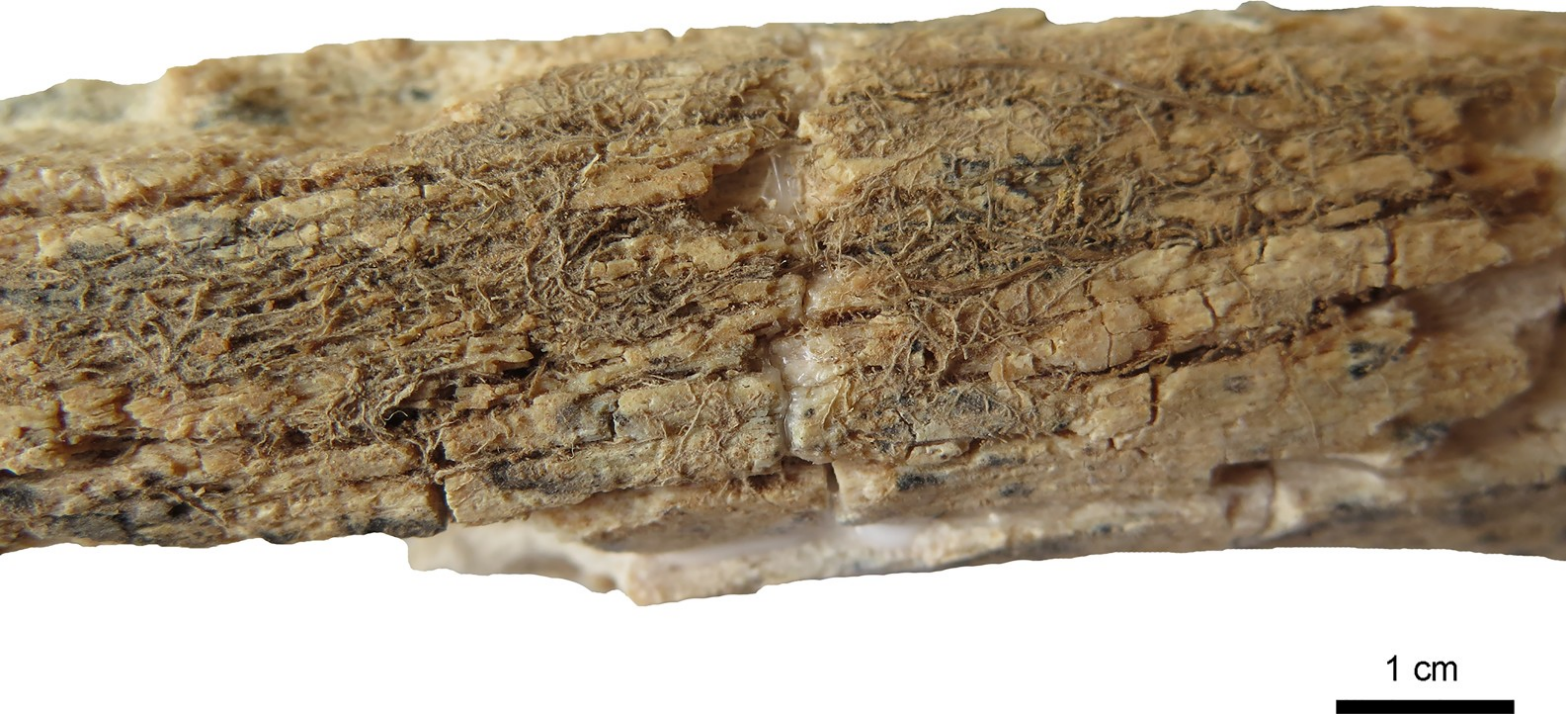
Detail of Fig 5. Roots attached as a dense net on the bone surface. (Photo: JG).

**Fig 7:**
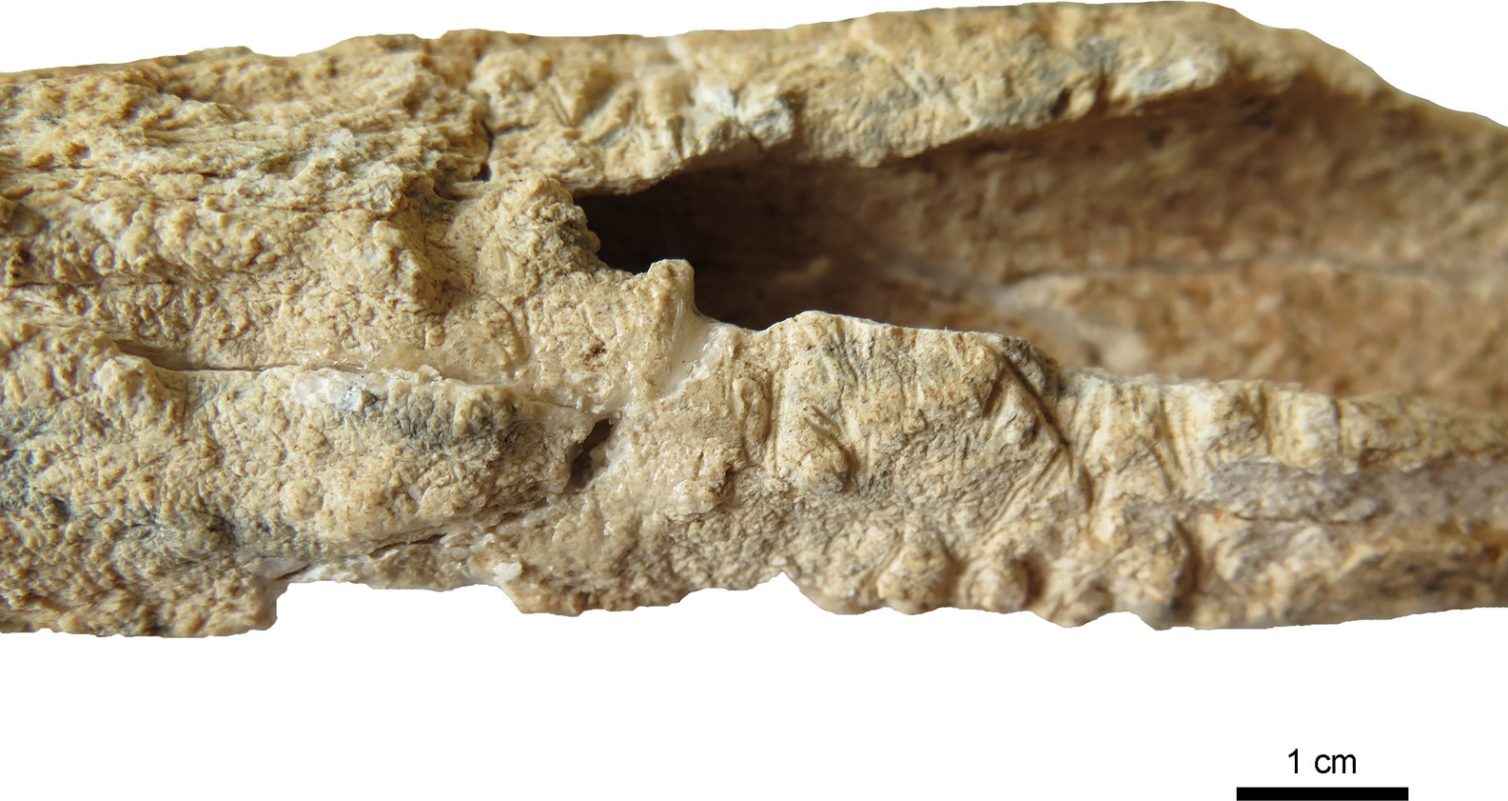
Dorsal side of the left femur showing destruction by rodent gnawing. (Photo: JG).

Taphonomic features include rodent-gnawing marks and remnants of roots on the fragment of the right scapula and very intense on the left femur (Figs 5–7). The surface of the femur is additionally changed by erosive processes due to water and stones. Remnants of roots are visible on the internal lamina of the right parietal too.

**Fig 8:**
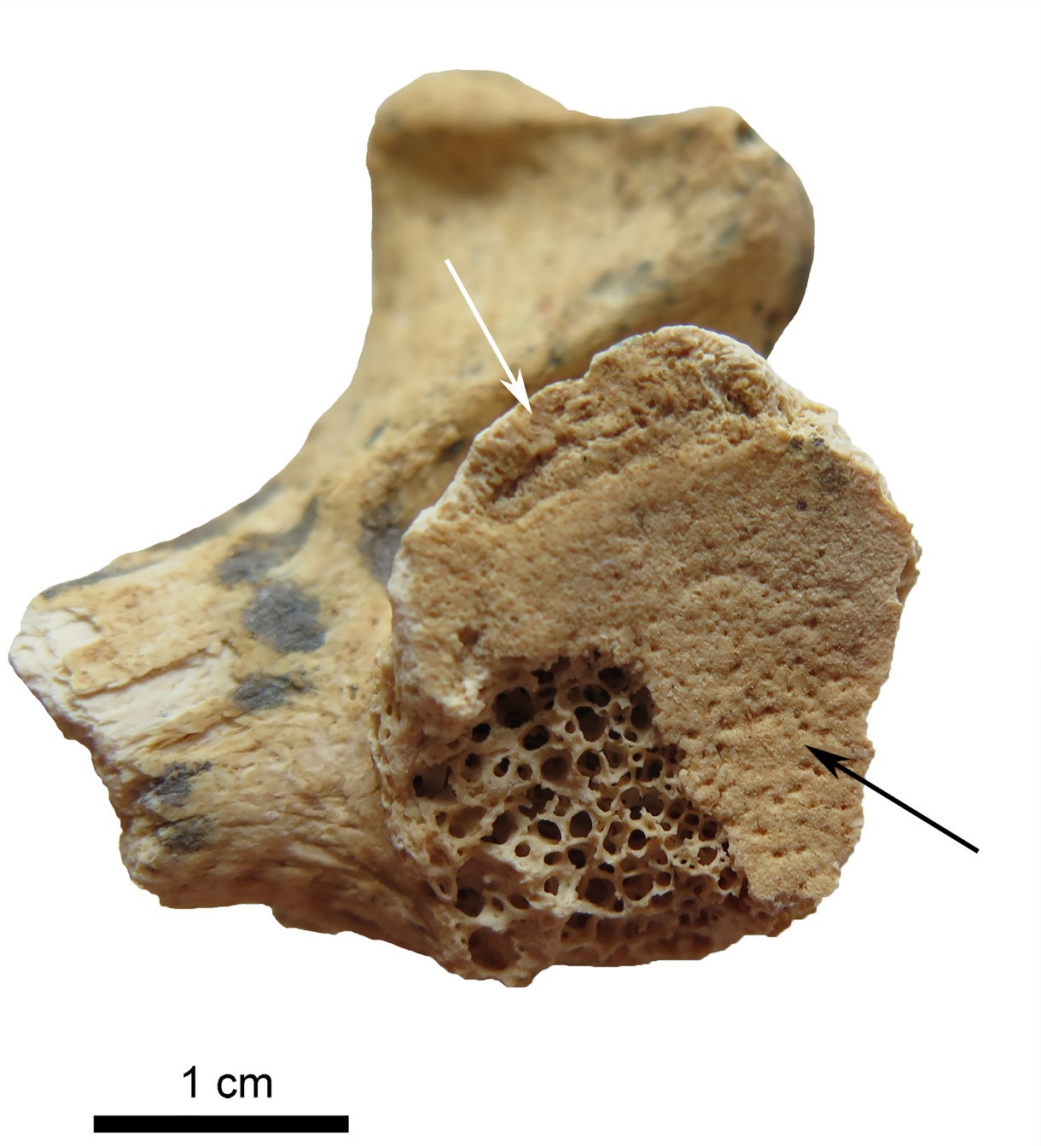
Atlas of the individual of Loc. C10:408. The left inferior apophyseal joint with small osteophyte of the rim (white arrow) and pitting of the surface (black arrow). (Photo: JG).

Joints of the spine are only scarcely preserved. Except the left lower joint of the atlas which shows pitting of the surface and subtle enlargement of the rim (Fig 8), all joint surfaces are without any pathological changes. The joints of the extremities (distal right *humerus*, proximal right *ulna*, possible right *patella*, proximal right *femur*, right *calcaneus*, small joints of hands and feet) are all without degenerative changes.

**Fig 9:**
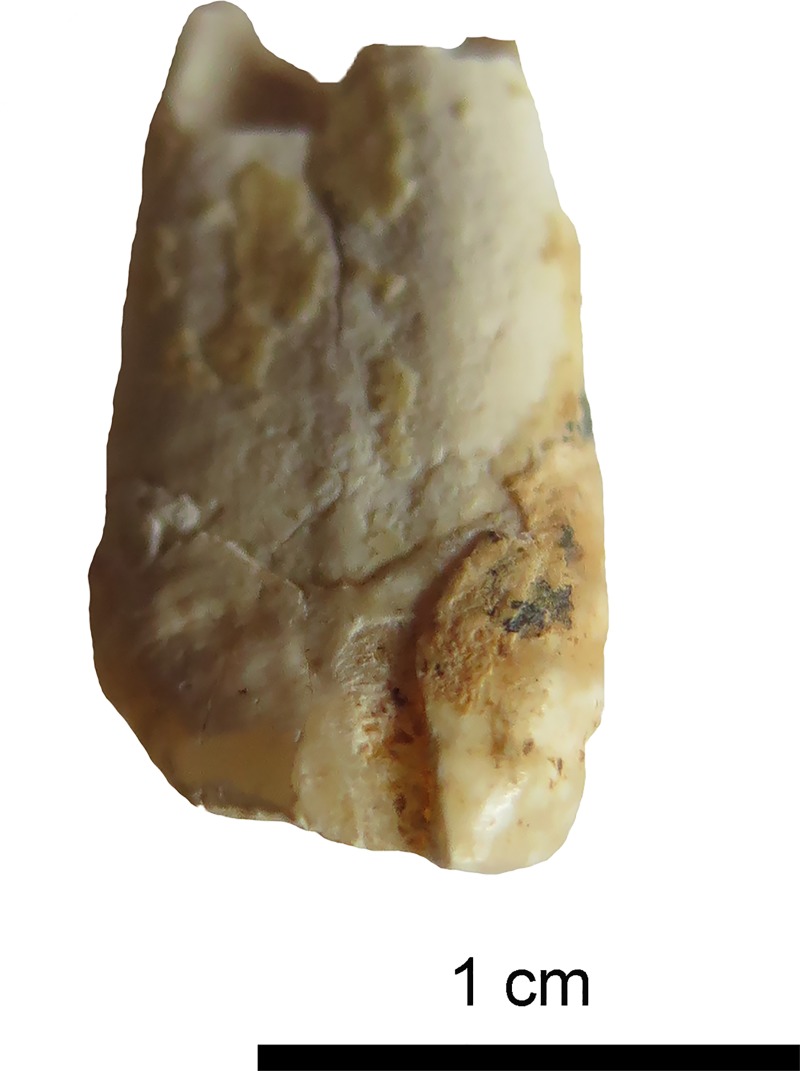
Tooth 23 with large chipping of enamel on the mesial side. Presence of calculus on the crown and neck. (Photo: JG).

**Fig 10:**
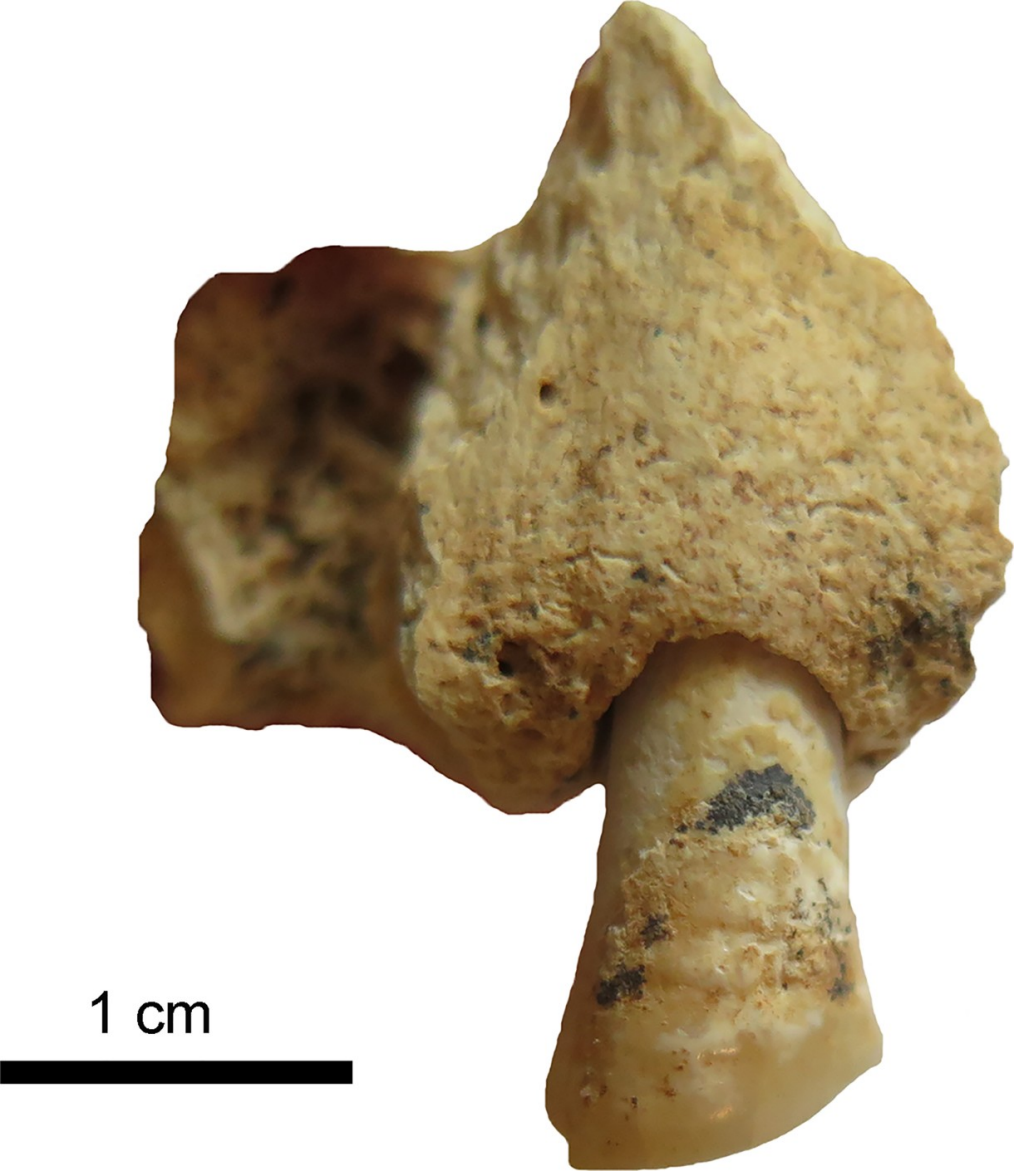
Tooth 23 with transversal enamel hypoplasia partly covered by calculus. (Photo: JG).

**Fig 11:**
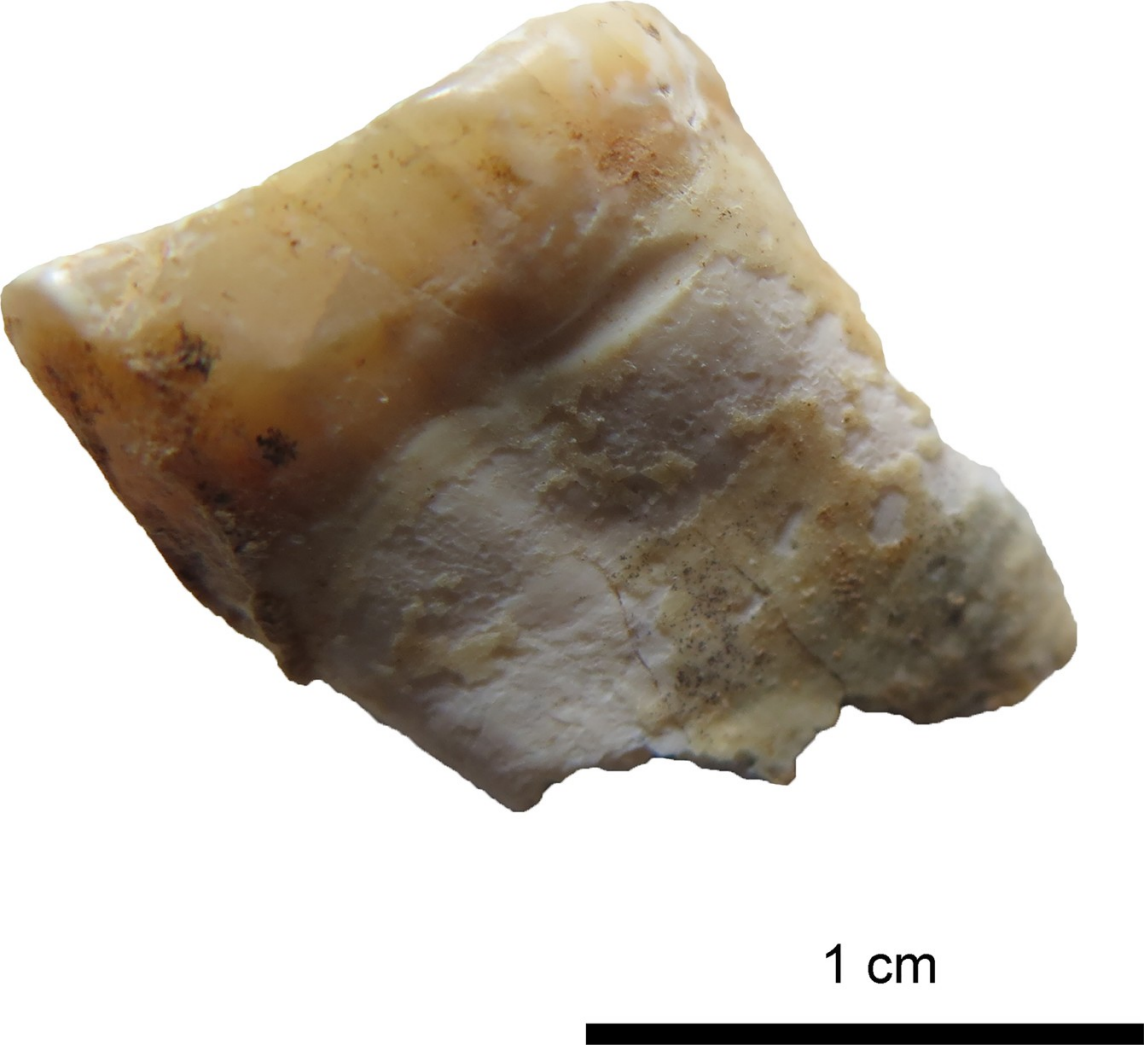
Tooth 27 with distinct interproximal grooving on the neck of the mesial side. (Photo: JG).

**Fig 12:**
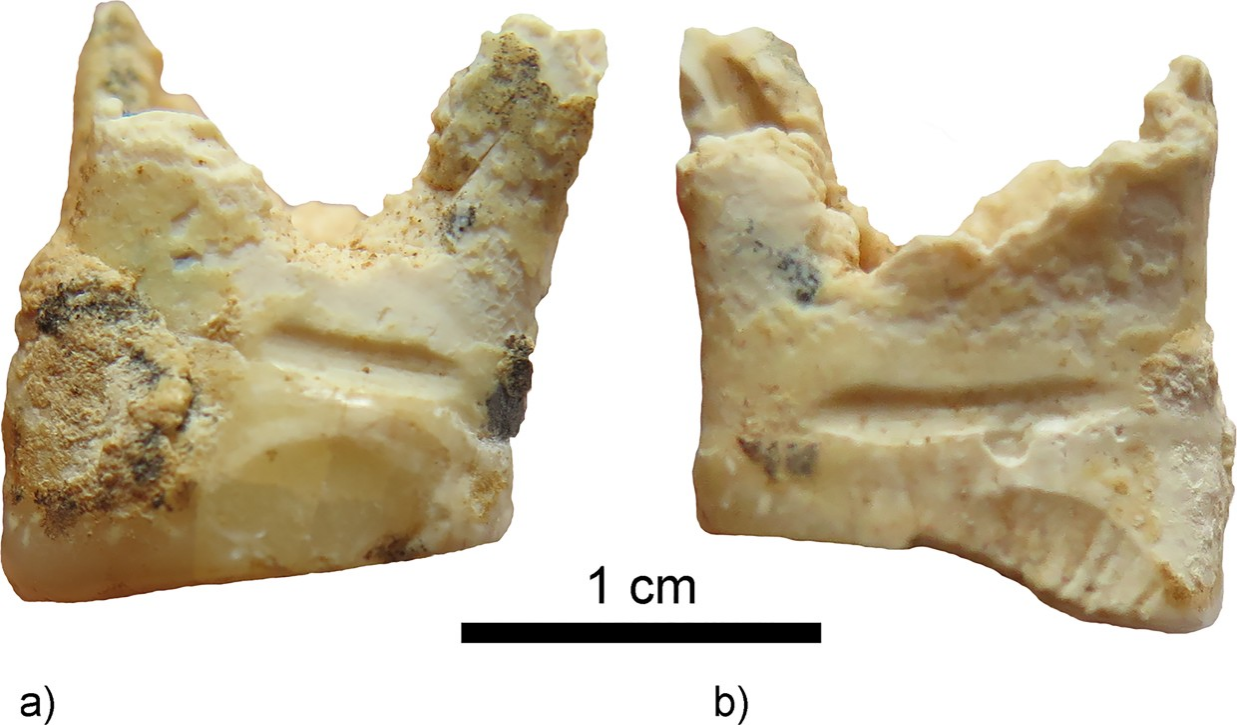
Tooth 26 with distinct interproximal grooving on the neck. a) on the distal side b) on the mesial side. (Photos: JG).

Only parts of the left *maxilla* and mandible are preserved, of the right side, only isolated teeth are present for investigation. No statements about periodontal diseases could be made due to poor preservation of the alveolar region. Apical processes seemed not to be present on the left jaws. Calculus is present on all of the teeth to a low to moderate degree (Grade I-II, after [65]) (Figs 9–12). There was no sign of carious lesions. When comparing molars and premolars, dental wear is more pronounced on the left than on the right side of the jaws. Severe dental wear is evident of the left incisors, canines and premolars (right side is missing). The distinct difference between wear of the anterior and posterior dentition suggests work-related use of the anterior teeth. Enamel chipping is present on 12 of 21 teeth, mainly in the posterior dentition. The chippings occur mainly singular from less than 1mm to 4x4mm in size (Fig 9). They might be due to hard substances within the food as they are predominantly affecting the molars and premolars and are not in the anterior region where it might be rather related to biting or work-related activities [66].

Linear enamel *hypoplasia* are present on 13 of 21 teeth in a moderate expression (Grade II, [65]) (Fig 10). This condition represents a disturbance of enamel formation, which is attributed to periods of malnutrition or infectious disease during the developmental period of teeth (see *e*.*g*. [67]). Their occurrence can be related to the ages of 6±1 and 12 years [68].

Signs of interproximal grooving as straight shallow grooves with a semi-circular diameter are visible on the lingual half of the mesial surface (4x1mm) of tooth 27 (Fig 11), on the distal surface (5x1.2mm) (Fig 12A) and on the mesial surface (7x1mm) of tooth 26 (Fig 12B). Teeth 18, 28, and 36–38 do not show interproximal grooving, the other molars are too poorly preserved, the area of interest mainly missing.

Concentrations of fine roots and a possibly acid milieu in the grave caused the poor preservation of collagen. Both aDNA analysis on tooth (27) and stable isotope analysis of carbon and nitrogen failed because of these conditions. Strontium isotope ratios (^87^Sr/^86^Sr) (Table 4) were obtained from the lower right third molar (48) of the adult individual (Loc. C10:408; 0.70812) and from the lower left second molar (75) of a 3-4yrs old child (Loc. C10:405; 0.70811), who was buried in the same room in the south-eastern corner [56] (S1 Protocol). Both values are identical, even within the measurement error. This indicates that the adult and the child procured their food and drink from resources in the same habitats during enamel formation. Assuming that the child did not migrate in his/her short life, both individuals can be considered to be of a local origin. This is confirmed by two, almost identical, ^87^Sr/^86^Sr ratios from rock hyrax (Procavia capensis), a local medium-sized terrestrial mammal (Table 4). In comparison to published data from the wider area, the values from Ba‘ja are at the lower range of the limestone formations of the Mount Carmel Area and Upper Galilee (0.7083–0.7086 [Kebara and Hayonim Cave]; [69]) and of the Basta individuals (0.7080–0.7082; [70]) (for the geographic location of all mentioned sites see S1 and S2 Figs and S2 Table). At first, this seems unexpected, since the location of Baʻja at the edge of Cambrian (541–485.4 mya), Ordovician (485.4–443.8 mya) and Cenomanian (99.6–93.6 mya) sandstone formations should have led to higher values than the younger Santonian-Turonian limestone area (about 94–83 mya) of Basta. However, the values corroborate earlier observations that there was no *in situ* supply of water close to the site. The next fossil water sources for Baʻja are at about 1–5 km to the east in the Naʻur-Fuhays/Hummar/Shuʻayb Wadi as-Sir limestone formation. It is also possible that rainwater, which might have been stored in the *siq*, functioned as a water reservoir [3, 71]. It can be suggested that this was the local value of the area, indicating that the food and water supply regimes included the adjacent limestone formations. A systematic isotope survey for reference samples can clarify this issue.

**Table 4:** ^87^Sr/^86^Sr ratios measured on LPPNB human teeth and on animal bones and teeth from Ba‘ja.

Context/ID	Species	Age (yrs)	Sex	Tooth body part	Sample ID	^87^Sr/^86^Sr	±2 SD
BJ16; Trench C10, Room CR 35, Loc. C10:405, double infant burial; Ind. II	human	3–4	indet	75	MA-172897	0.70811	0.00001
BJ 16; Trench C10, Room CR 35, Loc. C10:408, adult burial	human	25–35	male ?	48	MA-172898	0.70812	0.00001
Test Unit 2, 4126; BA’JA 4126_T_M	procavia capensis			tooth fragments	MA-190953	0.70815	0.00001
C12, Loc C12:18 Fz. 4025; BAJA 4025_T_M	procavia capensis			tooth fragments	MA-190954	0.70814	0.00002
C12, Loc C12:30 FN 4124; BAJA 4124_T_SC	equus africanus			bone	MA-190955	0.70829	0.00001

### Grave goods

The objects found in the grave can be divided in two main categories: items found inside the grave pit and objects embedded into the stone layer above the grave covering stone slabs (for all measurements see S1 Table).

#### Grave goods inside the grave

**Fig 13:**
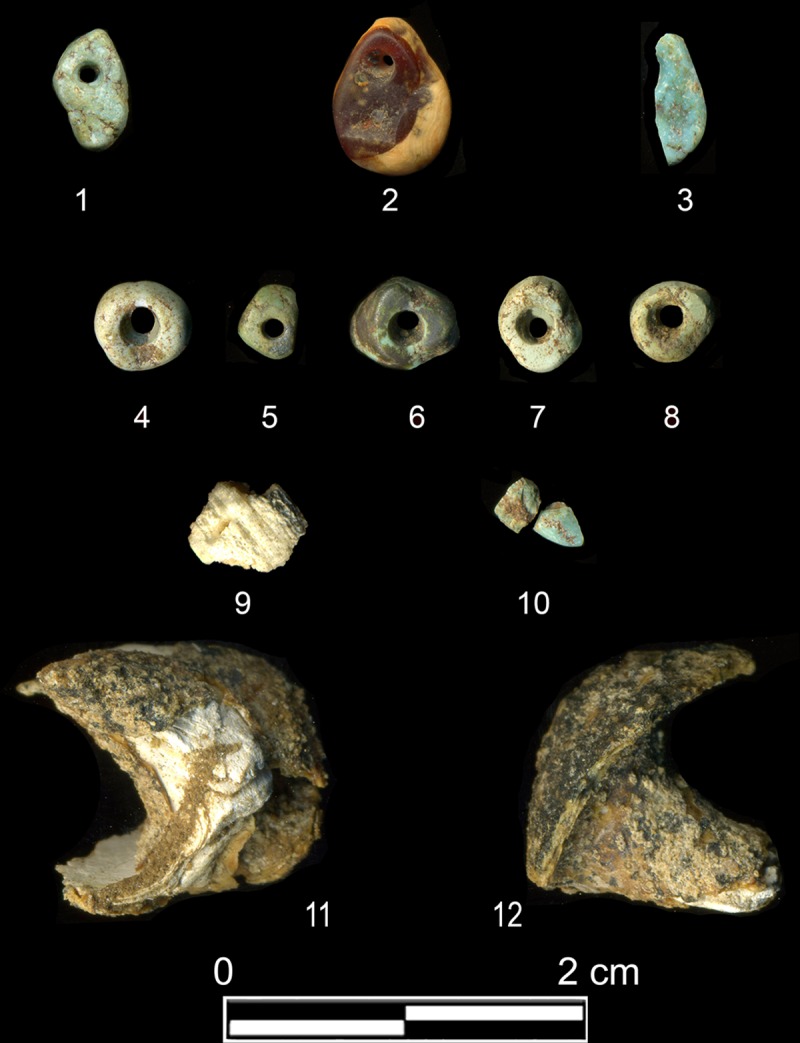
Beads from grave Loc. C10:408. 1, 4–6: turquoise; 2: carnelian; 3, 10: fragments of greenstone (amazonite?); 7, 8: turquoise (?); 9: fragment of shell bead; 11–12: two fragments of Conus sp. (Scan: HGKG; modified by HA).

**Fig 14:**
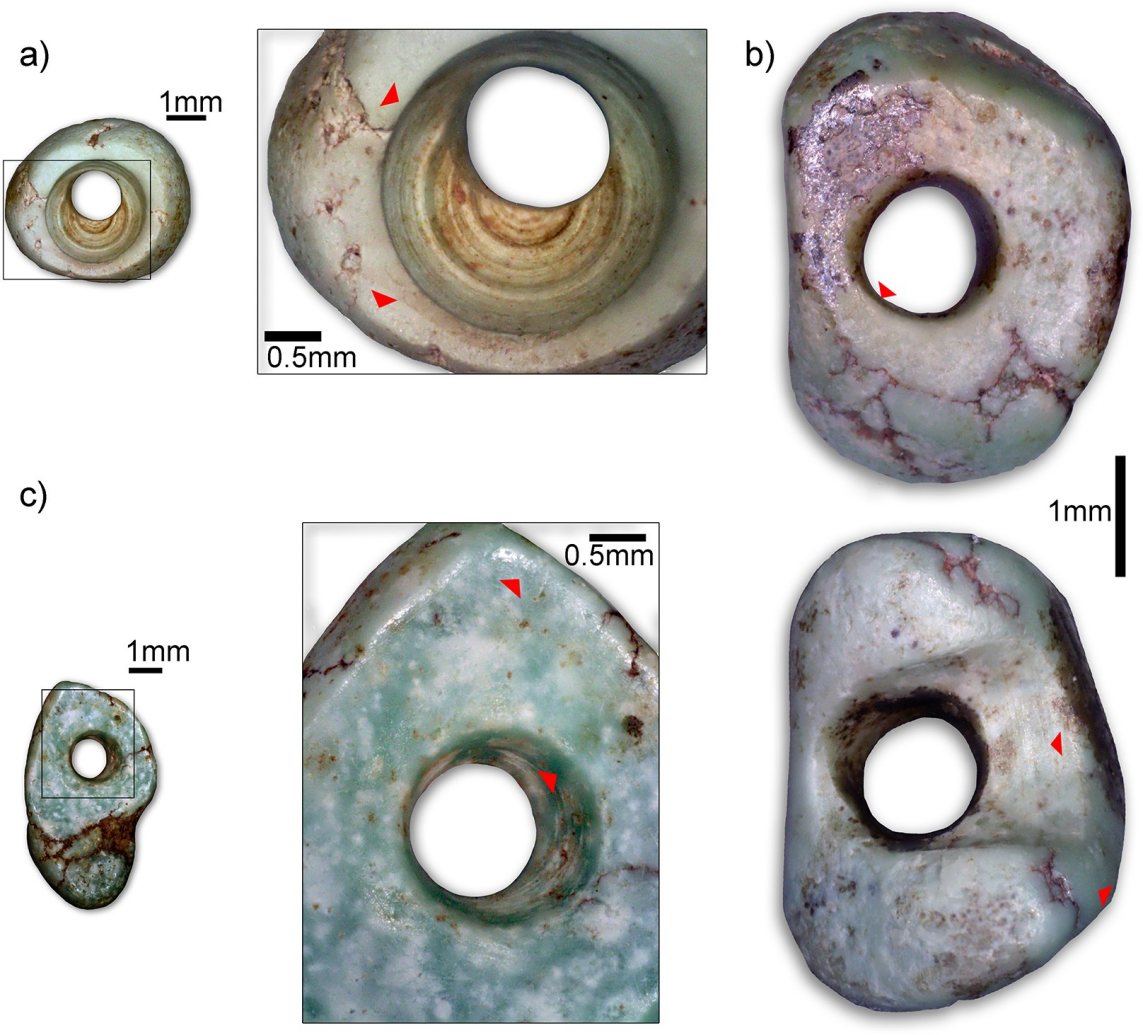
Close-ups of beads with different use-wear traces. a) Turquoise (?) bead N°8. Soft rounding of the hole edge (upper arrow), narrow and light polish restricted to some edges (lower arrow). See also the marked striations of drilling; b) Recycled tubular turquoise bead N°5. Drilling striations of a broken bead (upper arrow, lower photo), heavily smoothed and rounded edges and surfaces (lower arrow, lower photo, soft rounding of the “new” hole (arrow upper photo); c) Turquoise pendant N°1. Intense rounding of the edges with shiny polish (upper arrow), partial erosion of the drilling striation (lower arrow). (Photos: HA).

Around the left side of the head and on the chest of the individual were at least six isolated turquoise beads [72] and additional fragments of greenstone that may be amazonite (Fig 13). The beads were examined macro- and microscopically according to a use-wear approach [73–74]. They show use-wear traces all over the surfaces and with various stages of intensity: from moderately to heavily used and recycled items (Fig 14). A carnelian bead, shells and mother-of-pearl fragments were recovered from the sieved grave sediment.

**Fig 15:**
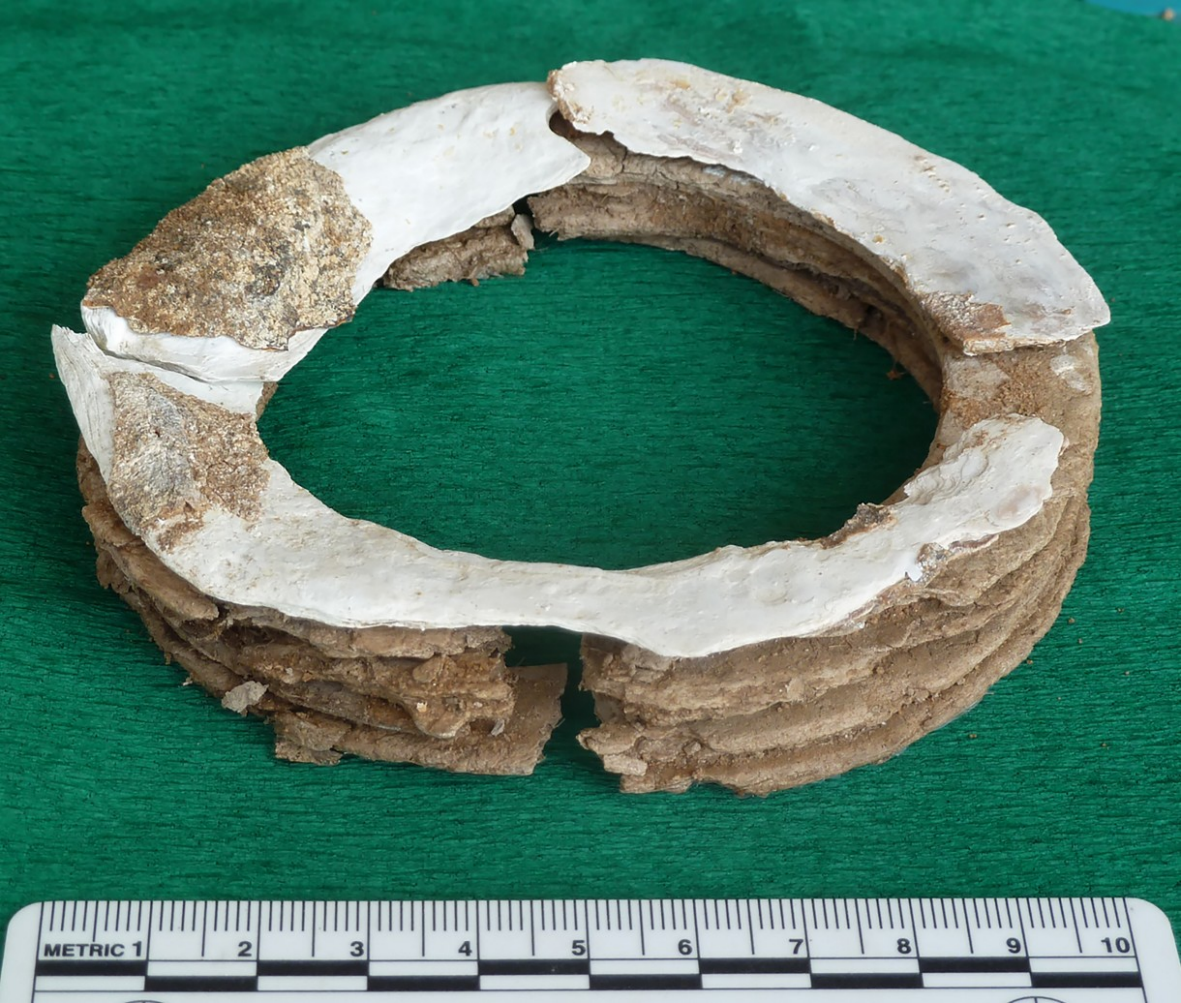
Composite upper arm ring of Loc. C10:408. This unique object was made of one mother-of-pearl ring and four marl (?) rings. It was worn on the left upper arm. Republished from ex oriente e.V. under a CC BY license, with permission from Hans Georg K. Gebel, 2017.

On the left arm, the individual wore a composite upper arm ring made of one complete mother-of-pearl ring attached to four layers of marl (?) rings (Fig 15). On the right upper arm, he also wore a mother-of-pearl ring made of several pieces. Judging from the inner diameter of both rings (±70mm), the buried person was not very corpulent, but rather gracile. The poor preservation of the human bone surfaces however precludes precise measurements.

**Fig 16:**
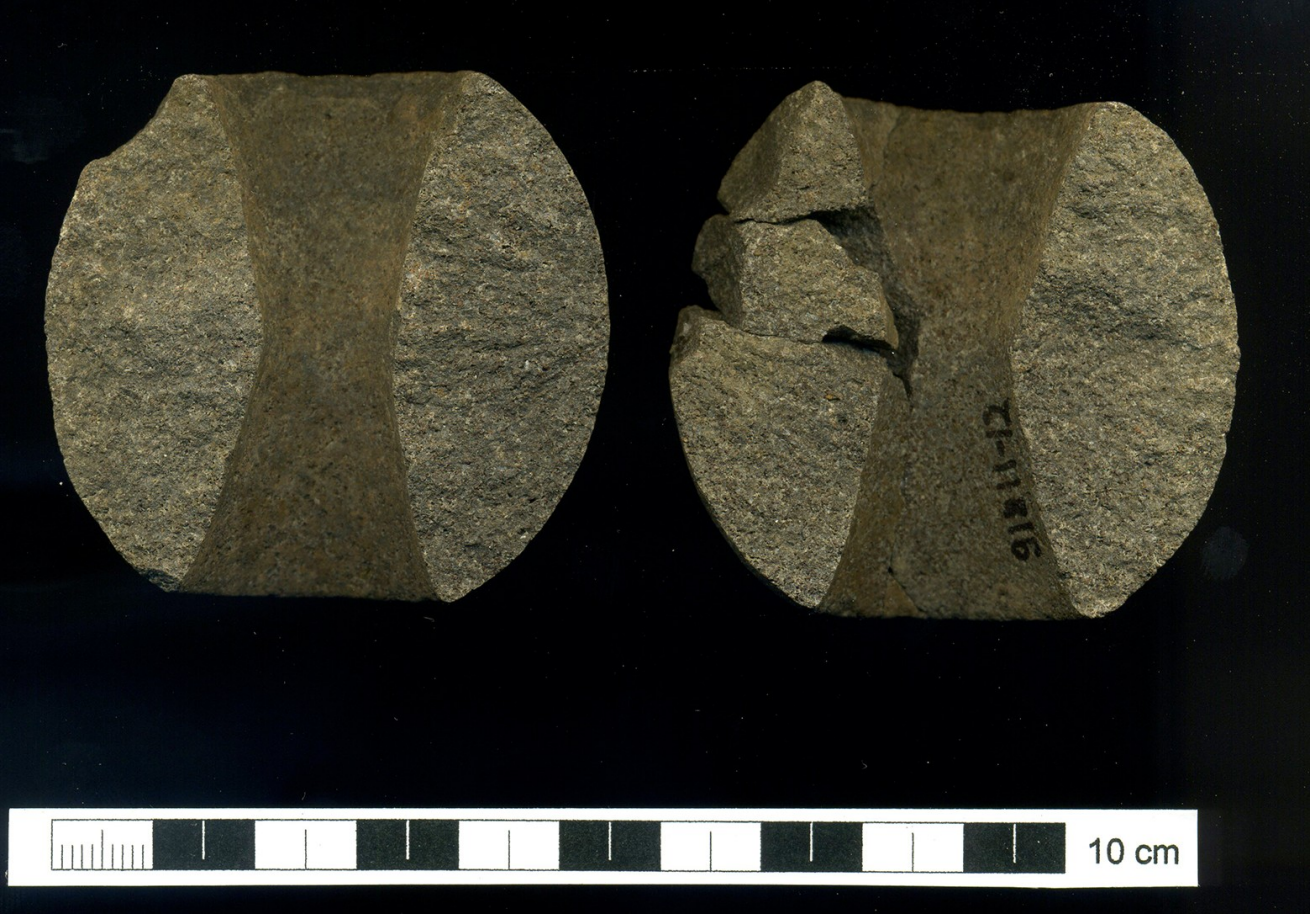
Deliberately broken macehead of igneous rock, Loc. C10:408. (Scan: HGKG).

The macehead next to the left shoulder was made of igneous rock. As mentioned above it was smashed *in situ* by a single high-energy blow (split in two halves with several fragments *in situ*; one fracture surface shows an impact point with radial scars) (Fig 16). Its height (53.0 mm) and weight (241.50 g) (see S1 Table) range at the upper end of Neolithic maceheads while the diameter (55.5 mm) is average [75]. A rather similar, but smaller and complete, item was discovered in the collective burial in area D (Table 5). Igneous rocks are not local. Between the fingers of the right hand, a red pigment mineral was found, but none of the bones showed clear red colouring, and the surrounding soil was not stained red either.

**Table 5:** Compilation of all burials of the LPPNB site of Baʻja.

ID/Location	Type	Number of Individuals	Sex	Position	Orient	CH	Red Pigment	MH	D	PP	BP	MOP arm ring	MOP amulet	Others	A	Reference
Loc. 26 Area D11/12/21/22	C, 0.65 m^2^, stone slab as grave cover (?)	3 juvenile- adult 9 infans I	mixed, indet	second-ary	?	X	X on bones and objects	1	1^†^^,^^‡^	9	“plenty”	0	1a, 1b	0	0	[58, 71]
Loc. C10:170/133, CR 34 Area C10	C, supra-floor, room as grave	2 infans II, 4 adult	1 female 3 male 2 infans indet	second-ary	1 skull facing east	Very few	X 1 piece	0	0	12	0	0	0	0	X	[59]
Loc. C10:152, CR 35 Area C10	C, 80x 70 cm Pit, stone slab as grave cover	2–3 adults, 1 juvenile 3–4 children (1 new-born)	1 male 1–2 adult indet Infans indet	second-ary	?	?	X on bones and grinding stone, pieces	0	1	4	>19	0	0	Grinding stone with red pigment, 1 hair slide, sandstone ring fragment, greenish pigment	0	[59]
Loc. C10:408, CR 35 Area C10	S, c. 1m^2^, cist grave, several coverings	1 young adult (25–35)	male ?	crouched, left side	SW-NE	Few	X 1 piece	1^†^	1^‡^	2^†^	>8	2^¶^	0	Pestle, stone vessel fragment, bone spatula	0	[56]
Loc. C10:405, CR 35 Area C10	Db, small round pit, c. 0,2 m^2^	2 infans I	1 female (a-DNA) 1 indet	sitting/ squatting	W-E, E-W, facing each other	0	0	0	0	0	0	0	0	0	0	[56]
Loc. C1:44/46, CR 36.1, Area C1	S, cist grave, c. 1m^2^, several coverings ≈ Loc. C10:408	1 infans II	Female	crouched left side	E-W	Few	X bones complete, 1 piece	0	0	0	>2500	0	1a	3 grinding stones with pigment, 1 red stained stone plate	0	This study
Loc. 5, TU 7, Area A	S, without grave pit, in rubble layer	1 adult 25–50 yrs	female ?	crouched, left, on back	N-S, facing S	0	0	0	0	1?	0	0	0	0	0	[59]

Type: C = collective, M = multiple, Db = double; S = single; Orient = orientation; CH = charcoal

Grave goods: MH = macehead, D = dagger, PP = projectile point, BP = beads and pendants, MOP = mother-of-pearl (a = with pierced appendices, b = without appendices), A = animal bones

†intentionally broken

^¶^ combined with 4 marl arm rings

‡ with burination; the burials are listed according to their location from north to south.

#### Grave goods embedded in the grave cover

**Fig 17:**
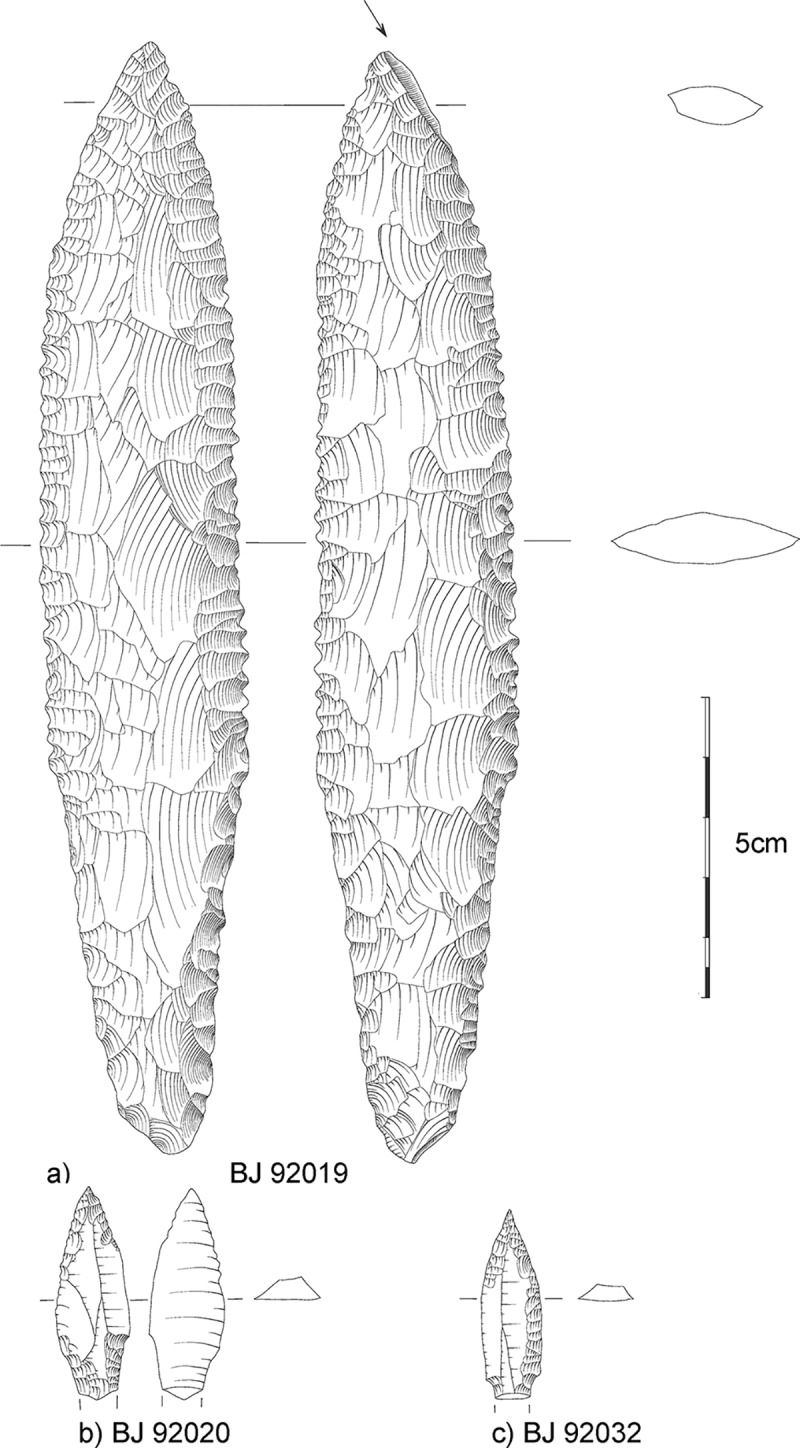
Flint objects of Loc. C10:408. a) the burinated dagger; b-c) two deliberately snapped projectile points. Printed under a CC BY license, with permission from Christoph Purschwitz, 2018.

A complete pressure-flaked serrated bifacial flint dagger was found in the western part of the grave cover (Fig 17A). Low-powered use-wear analyses confirmed the impact burination at the tip and discovered rounding of the edges of the serration as well as an overall polish along all the edges and even on the middle of the blade. The impact burination at the tip and the slight rounding of the serrated edges (for 9.5 cm from the top on both sides) could indicate single penetration of an object in a stabbing motion stopped by hard material (*e*.*g*. bone). The sharp unused serration at the base of the blade confirms that the dagger was definitely not used often as a cutting tool. The burination at the tip might have been caused by accidental or deliberate dropping on the floor. The uneven polish on many parts of the dagger may point to wrapping in a soft material or the use of such material during fabrication.

West of the dagger, a complete bone spatula and a cylindrical pestle of igneous rock were found. The pestle shows traces of use on its distal end. In the north-eastern part of the grave cover a rim fragment of a red sandstone bowl was uncovered. Furthermore, two flint projectile points of Byblos type were embedded in the cover (Fig 17B and 17C). Neither point exhibits any standard use-wear damage common on projectile points (*e*.*g*. diagnostic impact fracture or edge rounding), but they both have a broken tang caused by a snapping motion (which is very uncommon at the site).

## The M.A.R.L. Cube v1

**Fig 18:**
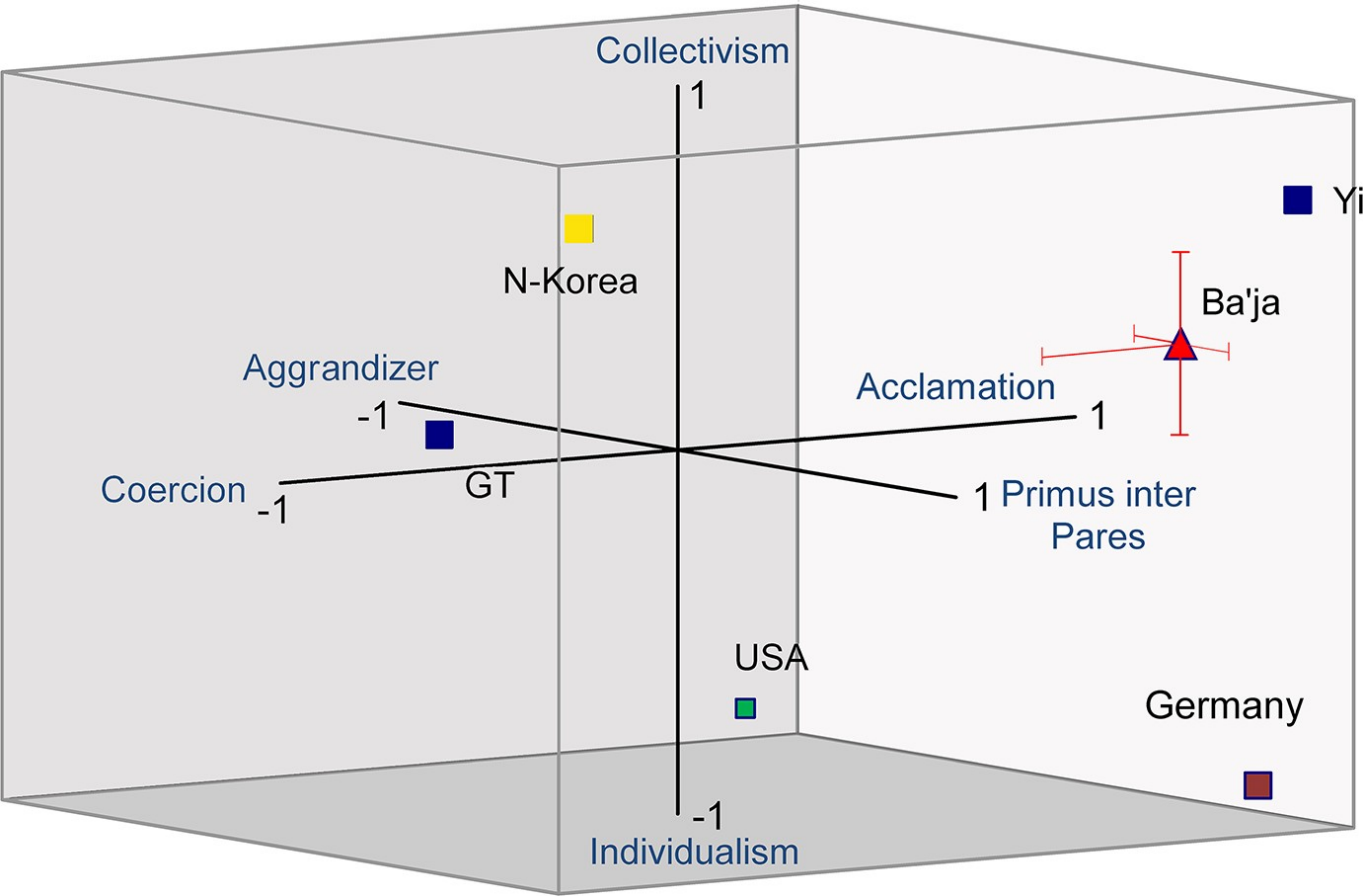
The M.A.R.L. Cube v1. For the description of social hierarchies, the leading agency (z-axis), the pathways to power (x-axis) and the socio-political ethos (y-axis) were considered. The position of Baʻja in the M.A.R.L. Cube v1 is given according to the median values of these three categories as elaborated in S1 Text. All other communities were positioned hypothetically as a contrast to Baʻja. A comprehensive analysis of the other communities according to the model remains a future task (e.g. GT = Göbekli Tepe [30]; Yi [10]). (Graph: MB).

Synthesizing the different aspects of socio-neurobiological, anthropological and archaeological research outlined in the theoretical introduction, and the empirical data that will be discussed below, the following M.A.R.L.-Cube v1 is suggested to describe different forms of leadership within their social contexts (Fig 18). The abbreviation M.A.R.L. stands for the main aim of the model: **m**odelling **a**nthropological **r**esearch on **l**eadership, v1 stands for version 1 (for the catalogue of questions and calculations see S1 Text and Tables A–D in S1 Text).

The M.A.R.L. Cube v1 respects the fact that the characteristic of the leader (1) is a crucial factor (z-axis: aggrandizer *vs*. *primus inter pares*). It also considers the relation of the social environment (2) to how power is achieved–either by self-motivated acclamation or by coercion–(x-axis: coercion *vs*. acclamation). And finally, (3) to whether communities are committed to a collective ethos with strong mutual relationships (y-axis: individualism *vs*. collectivism), *i*.*e*. that ideally the group’s need is more important than individual success, or to whether individualistic freedom is the ideal. With this three-dimensional model, communities can be localized in an area of probability even when some information is missing. Moreover, it allows the tracking of the development of a community from one area to another.

The three main factors: 1) leading agency 2) pathways to power and 3) socio-political ethos are studied according to the standardized questionnaire (S1 Text). For every factor, several subcriteria are studied and an estimated value is attributed on a scale between -1 and 1 in steps of 0.25 for every subcriterion, all of the latter are weighted equally. The calculated index is the median of all estimations for each factor. The mean absolute deviation from the median is indicated by the error bars. The median values of the main factors determine the position on the x-, y- and z-axis of the M.A.R.L. Cube v1. This comprehensive analysis should be considered as a first step in systematizing studies on emerging leadership. The application of the questionnaire to several case studies will allow a comparison of past and present forms of leadership systematically, despite the wide range of temporal and regional peculiarities. Since in prehistoric archaeology the only empirical data are physical remains, ethos can serve only as an indirect criterion. Aspects of material culture must be defined as evidence for ideas or social behaviors. The combination of material remains, anthropological data and imagery might allow a differentiation between represented ideas and daily practices in a qualitative investigation of the data (for such a multilevel approach see [77]).

The M.A.R.L. Cube v1 should be considered work in progress. It will prove to have its weaknesses and strength with further test cases and will probably require adjustments and refinements. It does not imply any evolutionary teleological tracks, but should promote a holistic understanding of different forms of leadership and of pathways to power.

## Discussion

In the following section the empirical data are discussed according to the three main categories elaborated in the M.A.R.L. Cube v1 (see above). The grave construction, the burial ritual and the grave goods have demonstrated that the buried person must have been outstanding in many respects [56] (Table 5). For an in-depth description of the various categories see S1 Text. The space “occupied” by the single burial is almost identical to collective graves of the site. Its location and construction differs little from other graves of the site following a more ancient tradition of cist graves discovered at el-Hemmeh [76] and nearby Shkārat Msaied [46]. However, it differs from other contemporary burials, single or collective, primary or secondary inside houses or in sub-floor channels at Beidha [78] or Basta [79]. The hermetic sealing of the grave pit with large stone slabs, gravel and white plaster as well as the bordering of the grave with a small wall is very similar to a lavishly decorated single child burial, recently discovered in the adjacent room south of Room CR 35. Covering the dead with such a tight sealing has generally been considered an effort to contain “the power of extraordinary individuals” [17]. Despite general similarities to other sites, the elaborate sealing of graves is unique at Baʻja so far. Moreover, the equipment is extraordinary, comprising exotic objects and possibly deliberately fabricated and destroyed objects for the burial ritual. Compared to other LPPNB burials in the region, until now, it is one of the most lavishly equipped burials. The differentiation of two sets of grave goods, deposited in two different events–inside the grave pit and in the grave cover–, also seems exceptional for southern Jordan, but a similar differentiation was observed in the above-mentioned child burial and at Kfar haHoresh [80]. In the following section, we will discuss the empirical data according to the three analytical fields of 1) characteristics of the leader 2) pathways to power and 3) characteristics of the community.

### Characteristics of the outstanding person (S1 Text, Tables A and B in S1 Text, S1 Table)

The burial ritual as well as the objects found inside the grave and in the grave cover provide important information about the social identity of the buried person. The size and shape of the bifacial dagger resembles the two daggers discovered at Ba‘ja in the adjacent collective Burial Loc. C10:152 and in the collective Burial Loc. 26 in D11/12/21/22 [58–59]. A medial fragment of a fourth item was found in the room fill above the grave in Area D. A similar dagger fragment comes from the LPPNB site of Basta. All daggers are of non-local raw-material and were obviously not produced at Baʻja, but rather probably in the south-eastern present-day arid steppes of Jordan or in the Negev. The lack of waste for the production of such daggers at Baʻja underlines the exotic character of this object [81]. A very similar burination on the tip of the blade has been observed on the dagger of the Grave Loc. 26 in Area D. It thus seems possible, that the burination was a deliberate action related to the burial ritual as some kind of devaluation of the object. However, the dagger of the collective Burial Loc. C10:152 had not been destroyed.

The damage to the dagger from Loc. C10:408 parallels the deliberate destruction of the macehead and the projectile points. The position and type of fracture indicate that the projectile points were intentionally broken from the shaft of the arrows. These damages and the lack of characteristic use-wear traces suggest that the points were not used in daily life. The location of the projectile points and the dagger in the grave cover, in contrast to the macehead, might point to a different meaning. Maceheads are rare during the LPPNB. Beside the above-mentioned item of the collective burial of Area D from Baʻja, a single basalt macehead has been reported from LPPNB layer of ‘Ain Ghazal [82]. However, they become more common during the Pottery Neolithic and Chalcolithic. Most of them were made of various types of limestones, basalt being exceptional. Many of them were broken in two halves and only a few were discovered in graves [75, 83].

All three items–the projectile points, the dagger and the macehead–are potential weapons and might have characterized the person as a hunter or warrior. They could also have been laden with a purely symbolic meaning related to social status. During later periods, maceheads became classical symbols of status, but whether this meaning can be transferred to the Pre-Pottery Neolithic remains an open question. Irrespective of their precise meaning, it seems that the power displayed by these items should be terminated after death. The microscopic use-wear traces on the dagger and the regular connection of daggers with burial contexts at Baʻja suggest that these rare objects were possibly exclusively used in burial rituals or as symbols of status.

The above-mentioned composite upper arm ring is unique so far (Fig 16). The mother-of-pearl ring of the right upper arm was composed of several parts. Both items were so fragile that it is hardly possible that they were worn during daily life, but they probably represented high status or prestige objects worn during lifetime at special occasions or offered after death.

The material of the beads corresponds to the increasing use of stone beads in general [73], and especially green-stones during the PPN [84–89], a trend that had already started during the Natufian [69, 90–94]. Turquoise and carnelian are exotic to the site. Raw material sources of turquoise are known in the Negev and Sinai [85]. The closest sources of carnelian were recorded in the Negev, Sinai and western Saudi-Arabia [93]. Carnelian bead production on a large scale at the oasis of Tayma in north-western Saudi-Arabia during later periods hints at close raw material sources as well [94]. If the raw-material determination of amazonite were confirmed, it would point to the Hisma in south-eastern Jordan [95]. Microscopic analyses of the beads showed that they were in use for a longer period of time. The homogenous distribution of the use-wear traces all over the surfaces indicates that they were enchained rather than fixed on clothes or other support. Whether they were offered by people participating in the funeral or whether they were personal items, remains an open question, but the position around the neck speaks in favour of the latter option. The handling of the beads over a long time and the recycling, points to the importance and value of these items. However, except for the upper arm rings, all the other objects were not exclusive to this burial, *i*.*e*. access to the raw-material or to the finished products, was not restricted to certain inhabitants of the settlement. Compared to the recently discovered child burial (Loc. C1:44/46) the number of beads even seems to be rather low.

The body’s position recalls the crouched position of one of the upper skeletons of the collective Burial Loc. C10:152 and the burial from the midden context TU 7, Loc. 5 [59] as well as the above-mentioned child burial in the southerly adjacent room CR 36.1 at Baʻja. Interestingly, neither the skull nor any other body part had been removed. This trait strongly differentiates the adult individual in Loc. C10:408 from the collective burials at Baʻja and also from many other individual or collective burials where fragmentation, segregation and re-deposition of isolated bones or body parts seemed to be common ([46, 96–97], see also [17]). The identity of this individual should not be merged with a “collective body of ancestors” (p. 188–189) [46].

Furthermore, the small diameter of the composite upper-arm ring, if worn during life, hints at a very gracile person. This physical trait might underline the special position, especially in an early farming community, where daily work-load probably required physical strength [98]. However, a preliminary notion on the late PPNB population of Basta suggested a “relatively gracile typus” too [79]. Only further analyses of all individuals from Baʻja and the results of the detailed analyses of the Basta individuals will show whether gracility is a typical trait of the local population. A preliminary analysis of the children of Burial Loc. C10:405 suggests rather robust body constitution. Transverse linear enamel *hypoplasia* indicates periods of stress (*e*.*g*. malnutrition or infections) during childhood, and might contradict ascription of a special status due to birth. However, *hypoplasia* is common in Neolithic communities [96–97] and might also evidence children whose immune system was efficient enough to survive these periods of stress. The chipping of enamel on some teeth hints at the consumption of very hard material, perhaps pistachio nuts or almonds. Rather weak attrition of the molars and the lack of caries contradict increased consumption of a diet rich in ground cereals, *cf*. [99]; very few incidences of caries (2 of 22) were also observed at Basta [96]. Interproximal grooves on two of the molars might hint at cleaning teeth with a thin stick made of bone or wood. Whether the differential wear of frontal and posterior teeth reflects a working task exclusive to this person or characterizes the habit of a special group has to await the analyses of the whole corpus of individuals. Neither chipping nor interproximal grooving has been reported from former investigations at Basta or Baʻja [96]. The results of the strontium isotope analysis suggest that the buried person lived directly at or close to the Neolithic site of Ba‘ja.

To sum up, empirical data for the character of the outstanding person tend to speak in favor of a possibly local, corporate prototypical leader, who was clearly displayed after death as a member of the local community following the structure of common collective burials. The material objects, burial ritual and grave construction tend more to a representation as *primus inter pares*. However, the reclaimed space, the efforts in grave construction, the single burial, and the display of exotic personal items inside the grave reduce the median of all sub-categories from a purely *primus inter pares* type (= 1) towards the aggrandizer type with a median of all subcategories at **0.38±0.17**. The distinct spatial segregation suggests that he should be remembered as an individual in the collective memory.

### Achieving excellence: Acclamation *vs*. coercion (S1 Text, Table C in S1 Text)

Achieving supra-regional power by physical coercion has been suggested as a prime mover for establishing chiefdoms [6]. Empirical data from the early Neolithic of the Near East has been interpreted in both directions: absence or presence of supra-communal conflicts (*e*.*g*. [100] *vs*. [19]). No evidence of physical force has yet been observed on the individuals from Baʻja. However, skull traumata and one lethal injury were reported from other contemporary sites: Basta (5 of 29 individuals; [96]), Wadi Shu’eib (3 of 17 individuals; [101]) and ‘Ain Ghazal (1 of 7 individuals, [82]). An in-depth discussion of the possible reasons for these injuries (interpersonal conflicts, accidents, intra- or inter-group conflicts, or both?) is out of the scope of this paper. However, physical force is only one aspect of human aggression. A lack of evidence for injuries does not exclude subtle means of suppression such as social or economic deprivation.

There is no imagery showing the position or social role of the individual, but both categories of grave goods comprise potential weapons. All of these items were deliberately destroyed or showed damage which meant at least a partial loss of value. The micro-traces of the dagger indicate that it was made for status, representation or ritual rather than for daily use. As suggested by Gebel [3] there is no clear evidence for “(aggressive) institutional forces”.

With the burial of that person, his power should be terminated too. Prestige or status symbols were deliberately destroyed and not transferred to a successor (for a similar praxis see [102]). As mentioned above, the hermetic sealing of the grave also hints at an effort to terminate or control the possible (dangerous) power of the deceased [18]. This might suggest that the outstanding position was acquired through personal skills or properties rather than by heritable institutionalized succession. Future aDNA analyses might help to solve the question whether status was due to familial succession but, for now, there is no sign of ascription of an outstanding status due to birth.

Furthermore, the individual was buried shortly after death, given that most of the bones were still in an anatomically correct position. Drying by mummification cannot be excluded, but the lack of gnawing traces from carnivores means that it is improbable that the body was laid out for drying. In contrast to other early Neolithic sites of Basta and Göbekli Tepe [97, 103], no cut marks have been observed, but they might partly be obliterated by poor preservation of the bones’ surface. Whether the shallow white monticule formed by the grave served as a point of reference or memory is unclear. The physical presence of this person after death was definitely less significant than of selected persons of other MPPNB and LPPNB sites, whose skulls were retrieved after death, plastered and exhibited for some time. Even after reburial, groups of skulls served as points of reference [104–107]; for a recent summary on that topic see [31] *vs* [36]). This difference can be interpreted in two directions: prestige had been acquired due to personal traits of behaviour and ended with death; or the individual only played an ascribed role during life-time in an institutionalized rank-system, being replaced after death by someone else, *i*.*e*. his function within society made him outstanding. Taking into account the evidence from the grave goods, which were neither exclusively used, nor given over to a successor but destroyed, the first interpretation of acquired prestige or status seems more probable. However, the on-going studies of the above-mentioned lavishly decorated child burial Loc. C1:44/46 may require reconsidering these conclusions in the future.

Not a single fragment of the otherwise ubiquitous sandstone rings was found in the burial Loc. C10:408, clearly separating “economic” commodities from other rare (prestige) objects [26, 108]. Only in the adjacent collective burial Loc. C10:152 was a fragment of a sandstone ring discovered [59]. It can thus be suggested that it was not the accumulation of economic wealth that contributed to the high ranking position of this individual; at least, during burial ritual, the displayed ideology did not aim to demonstrate accumulated wealth but rather rare, highly symbolically laden objects. The decoration with exotic beads (or the giving of beads) should probably indicate access to, if not active participation in, a supra-regional exchange network [109]. The different intensity of use of the beads might indicate that their exchange was used to signal and possibly reinforce social relationships. In contrast to the nearby sites of Shkārat Msaied [89], Beidha [110] and specialized amazonite workshops in the Hisma [95], no bead workshop has yet been identified at Baʻja and micro-drills are very rare at the site [81]. However, the unfinished perforation of some beads, fragments of greenstone beads, and pieces of carnelian raw material might evidence some local production.

The supra-regional–possibly down-the-line–exchange networks represent very old relations from the Red Sea to the northern Levant [73–74, 109, 111]. Non-local raw materials indicate exchange with eastern modern-day steppe areas, the Negev and Sinai and possibly western Arabia. The origin of igneous rock has not yet been determined. Whereas shells were exchanged in large quantities, green stone and carnelian beads represented rare items, even during the PPN [73, 85–86, 89, 112]. During the LPPNB, non-local flints declined considerably. This might hint at reduced access to these exchange networks [81] or shifted acquisition patterns for flint raw materials. Although access to exotic objects was not confined to one individual or group and although the spectrum of exotic materials increased, the general trend of reduced non-local raw materials might have contributed to enhancing prestige of those who still had access to these networks [81].

Though most of the recorded items seem to be of profane use, it cannot be excluded that some of them were especially produced for a funeral function with a special ritual meaning. The few use-wear traces on the dagger and the fragility of the arm rings might point in that direction, but it seems premature to decide on “religious” or profane status of the individual, *cf*. [4].

Considering the evidence of access to power, clear evidence for physical coercion is lacking. It seems probable that excellence was attributed as a result of personal behavior or traits. Any conclusion about the importance of familial relationships remains highly speculative since information on genetic relations is mainly lacking. Only further anthropological analyses might clarify familial relationships between the lavishly decorated burials in Area C and the other collective burials. The median value for the analyzed data ranges at **1 ± 0.35** showing a strong tendency to acclamation or ascription of a social position rather than achieving power by coercion.

### Ethos of the community: Individualistic *vs*. Collective (S1 Text, Table D in S1 Text)

Generally, the community of Baʻja seems to be rather homogenous, yet without strict canonization. At least during its final occupation, the agglutinated rooms appear as one confined entity bordered by the deep gorges of the siq. Despite complex use biographies and constant changes in spatial layout, domestic buildings did not differ categorically from one and another [71, 113–114]. Walls were so close to each other that daily life probably became a challenge. So far, neither special or communal buildings nor communal monuments have been discovered. Except for the parallel walls that were sloping in north-south direction on the southern slope of the settlement in Area F [71], no terracing like that of Basta [115] has been recorded so far.

The distribution of objects seems to be rather homogeneous, too, so that the production of sandstone rings has even been termed “community specialization”. Nonetheless, some groups seem to have had access to better flint resources and were more skilled in flint production [81].

In the three collective burials of Areas C and D, individualism was annihilated and individuals (if they have been conceived at all as this) were merged into groups of dead. The contrast between collective and single burials might parallel the development observed in the central and southern Levant from a group-oriented ideal to the veneration of single adult individuals [4, 43, 116–117]. However, it is not only in the ritual sphere that increasing segregation is observed. Also in economic and social relations, relational corporate identities seem to dissolve due to increasing specialisation [19].

Evidence for doctrinal canonized symbolism is lacking in the southern Levant which is in strong contrast to Northern Mesopotamia [19, 37, 53, 117]. In daily practice things were repeatedly done in the same manner. For example, sandstone rings that were produced *en masse* at Baʻja followed a general idea and had a standardized *chaîne opératoire*, despite some variation in size and style. Similarly, burial rituals adhered to a general pattern of grave construction, but not one grave is identical to the other. For now, only two types of objects have been identified as highly standardized: the daggers and the mother-of-pearl ring spacers for small children [26, 58, 118–119]. Both items seem to convey certain identities.

It has to be emphasised that this broad outline still lacks meta-analyses of anthropological data. Even though many burials have been excavated in the southern Levant, a comparison of kinship, *e*.*g*. [70, 120], health status, mobility or diet on the basis of anthropological data has to await future research. Any conclusion about genetic relationships concerning social differentiation, such as inheritance, “lineages” or even “tribes” or “clans” remains highly speculative.

Whereas the median of the material records from the settlement and architecture show no clear trend **(0.0±0.42)**, the display of boundaries mirrors a strong collective identity **(0.75±0.25)**. The term corporate *sensu* Gebel [19] is avoided here since it implies confined corporate identities, which is not necessarily the case in the opposition between individualistic and collective ethos. Overall, the values for the group ethos tend to the collective side **(0.25±0.25)**.

## Conclusion

The summary of the results of the systematic analyses allows us to conclude that the individual buried in Loc. C10:408 was probably a local, outstanding person represented after death according to the structurally same burial ritual as other group members, but definitely set apart from the ordinary by the elaboration of the grave construction and the presence of exotic and technically sophisticated objects and materials (Fig 18). This individual was interred and kept in the collective memory *sensu* Assmann [121] as *primus inter pares* using structurally the same burial rituals and colour symbolism as in other graves. The deliberate destruction of symbolic grave goods of physical force hints at the termination of achieved prestige or status rather than an inherited status. The segregation from the other collective burials and the occupation of space similar to group burials point to an outstanding authority. This is in line with the suggested development from corporate identities during the middle PPNB to the increasingly extraordinary status of single individuals during the LPPNB ([116, 53]; see also [4, 43, 46]). The ongoing research on “Household and Death” at Baʻja [56] will possibly clarify whether the emulation of “the general corporate identity regime [had] started with ‘sub- or peer-identities’ becoming ‘non-corporate’” (p.70) [19].

For the LPPNB of Baʻja, we suggest that single individuals achieved prestige or status or both on the one hand by their access to exotic items, probably through ancient networks of equally influential persons in other communities of the southern Levant. They still had access to exotic items even though there was an overall reduction of imported material [81]. They were possibly the beneficiaries of increasing territorialism [3, 53] as intergroup conflicts might have enhanced their authority as leaders [3, 20].

On the other hand, he sought support in his local group by prototypicality, which is a characteristic of group-oriented chiefdoms [22] and of relational communities [19]. The position of the grave reflects this tension in a similar way: It was spatially segregated, but in the same room as the collective burial Loc. C10:152 and the double infant burial Loc. C10:405 [56]; close proximity to other group members should be maintained.

The outstanding person from Baʻja thus incorporates elements of both types of leaders–corporate and individualistic–in an otherwise increasingly heterarchical community. Neither a costly display of accumulated things nor a display of physical strength played a decisive role in the burial ritual; other criteria, such as personal skills, behaviour, or traits, must have been decisive. Future anthropological investigations will possibly help to refine our understanding of the reasons for the social segregation and excellence of this individual.

## Supporting information

S1 Table List of grave goods of Loc. C10:408. (DOCX)

S2 Table Means of ^87^Sr/^86^Sr-isotope ratios from different locations in the Levant (from south to north). (DOCX)

S1 Fig Geological setting of major Pre-Pottery Neolithic B sites in southern Jordan. Map designed by C. Purschwitz based on compilation of data from [122–128]; printed under CC BY license, with permission from C. Purschwitz, 2019.
(TIF)

S2 Fig Comparison of ^87^Sr/^86^Sr-isotope ratios from Baʻja with data from other geological settings in the Levant. Plain diamonds represent means; error bars represent the 95% confidence intervall for each site. Red: samples from Baʻja analyzed in this text; pinkish: (Pre-)Cambrian sandstones and granitic formations of Wadi Rum; blue/green: limestone formations southeast of Baʻja / in the Mount Carmel Area and Upper Galilee; orange: Quaternary basalts and limestones; brown: travertine deposits. Data sources: [69–70, 129–130]. (Graph: MB/CK).
(TIF)

S3 Fig Probability ranges of calibrated radiocarbon dates. (PDF)

S1 Text Analytical questionnaire for the Burial Loc. C10:408. (DOCX)

S1 Protocol Sample preparation for strontium isotope analyses. (DOCX)
