# Supplementary material for: Burying power: New insights into incipient leadership in the Late Pre-Pottery Neolithic from an outstanding burial at Baʻja, southern Jordan
Source: PLoS One. 2019 Aug 28;14(8):e0221171. doi: 10.1371/journal.pone.0221171 (PMC6713438; doi:10.1371/journal.pone.0221171)
Supplement: S1 Table — (DOCX) [file pone.0221171.s001.docx]

**S1 Table. List of grave goods of Loc. C10:408.**

| **Item,**  **Field number**  **(Fig reference)*** | **Location** | **Description, raw material, measurements**  (mm; l length, h height, w width, th thickness, d diameter, med medial, prox proximal, dis distal, pres preserved) |
| --- | --- | --- |
| flint dagger  92019  (Fig 17a) | embedded in upper sealing of grave | pressure-flaked dagger with impact burination, fine edge denticulation outside hafting/ prehension area; flint, complete  l 183.4; w max 33.3; th prox 7.5; th med 7.8; w dist 6.7; weight: 66.36g |
| bone spatula  95014 | embedded in upper sealing of grave | bone spatula from rib; complete, broken during excavation; l. 148.3; w med 18.1; w dist 19.7 |
| 2 projectile points  92020/ 92032  (Fig 17b-c) | embedded in upper sealing of grave | 92020: tanged arrowhead (tang broken); flint  l pres 30.6; w max 9.6; weight: 0.77g  92032: tanged arrowhead (tang broken); flint l pres 35; w max 11; weight: 1.2g |
| stone pestle  91813 | embedded in upper sealing of grave | cylindrical pestle, basalt?/ igneous rock; flat working face; proximal: old fracture  l 119.0; w max 54.0; weight: 625.80g |
| stone bowl  96804 | embedded in upper sealing of grave | rim fragm. of steep-sided bowl; light reddish sandstone  d 300?; th 10 below rim: 27 |
| macehead  91812+91811  (Fig 16) | inside grave, next to the left upper arm | mace head with biconvex outline and biconical perforation; basalt/ igneous rock; h 53.0; w 55.5; d central perforation 13.4; d outer perforations 25.2 and 24.9; weight: 241.50g |
| composite arm ring  91264  (Fig 15) | inside grave, around upper left arm | composite ring made of superposed elements: one carved mother-of-pearl ring and four rings carved from a layered (?) mudstone; fragility of mudstone (marlish/ clayish material) makes it likely that this ornament was applied only on the corpse (not worn); very fragile; poor preservation (soil acids, roots)  interior diam. +/- 69mm |
| mother-of-pearl arm ring  90400 | Inside grave, around upper right arm | ring of carved mother-of-pearl; poor pre­servation (soil acids, roots)  d interior +/- 70.00 (?) |
| 4 small mineral beads  90800  (Fig 13) | inside grave, neck and head area | 1 unfinished turquoise bead; l 6.75, w 3.85, th 1.89  1 broken turquoise bead, l 3.85, w (?) th (?)  1 turquoise flat semi-cylindrical bead, d max 5.12, h 2.90  1 turquoise flat cylindrical bead, d max 4.86, h 2.98  1 fragm. of greenstone |
| 4 small mineral beads  90803  (Fig 13) | inside grave (from sifted grave fill) | 1 turquoise flat cylindrical bead, d 5.01, h 2.26  1 greenstone facetted bead, l 5.37, w 6.05. th 4.74  1 carnelian bead, pendant-shaped, l 8.66, w 3.40, th 3.32  1 bead from sea shell heavily leached; shape (?); > 6.01 x 5.51  2 fragm. of amazonite/greenstone? |
| shell fragments  90802  (Fig 13.11-12) | inside grave, left part of chest | 1 Conus sp.; heavily leached; <15mm  1 (?), heavily leached; <16mm |
| shell fragments  90804 | inside grave (sifted material) | small and tiny shell fragments, affected by soil acid/ leached |
| mother-of-pearl fragments  90407 | inside grave (sifted material) | small and tiny mother-of-pearl fragments, affected by soil acid/ leached |
| “red mineral”  97902 | inside grave, between finger bones of right hand (gripping the left upper arm), holding it intentionally (?)/ reached this position after decomposition? | mixture (?) of red mineral, lime inclusions and fossilized material |

* Compilation and measurements of grave goods and objects embedded in the grave cover by HGKG.
