# Supplementary material for: Burying power: New insights into incipient leadership in the Late Pre-Pottery Neolithic from an outstanding burial at Baʻja, southern Jordan
Source: PLoS One. 2019 Aug 28;14(8):e0221171. doi: 10.1371/journal.pone.0221171 (PMC6713438; doi:10.1371/journal.pone.0221171)
Supplement: S2 Table — (DOCX) [file pone.0221171.s002.docx]

**S2 Table. Means of ^87^Sr/^86^Sr-isotope ratios from different locations in the Levant (from south to north).**

| **Name** | **Geographic locaction** | **Geological formation** | **Species** | **Mean (n= number of samples)** | **Confidence intervall (95%)** | **Reference** |
| --- | --- | --- | --- | --- | --- | --- |
| Ayn Abū Nukhalya (Wadi Rum) | 29°33'21"N 35°24'32"E | (Pre-) Cambrian sandstones | local wild animals (*lepus, ibex*) | 0.708393  (n=6) | 0.000036 | [129] |
| Ma‘an Plateau | 30°01'13''N 35°28'07''E | Cretaceous limestones | Local wild animals (snail shell, *laudakia stellio, rattus norvegicus*) | 0.708115  (n=4) | 0.000100 | [129] |
| Basta | 30°13'39"N 35°32'00"E | Santonian-Turonian- Cenomanian limestones and sandstones | Human | 0,708157  (n=30) | 0.000017 | [70] |
| Azraq | 31°49'N 36°48'E | Quaternary basalts and limestones | *gazella sp.* | 0.707935 (n=6) | 0.000107 | [69] |
| Wadi Hammeh 27 | 32°27'59''N 35°36'36''E | Pleistocene travertine limestone deposits | *gazella gazella* | 0.709278 (n=5) | 0.000210 | [69, 130] |
| Kebara Cave | 32°34'25''N 34°58'07''E | Cretaceous Limestones | *gazella gazella*,*vulpes vulpes*, *felis sp.* | 0.708462 (n=9) | 0.000081 | [69] |
| El Wad | 32°40'14''N 34°57'55''E | Albian–Early Cenomanian Limestones | *gazella gazella*,*vulpes vulpes*, *panthera sp.* | 0,708407 (n=9) | 0.000028 | [69] |
| Hayonim Cave | 32°55′18″N 35°13′06″E | Cretaceous limestones | *felis sp., meles meles, gazella gazella* | 0,7083725 (n=8) | 0.000057 | [69] |
| Mallaha | 33°04'59''N 35°34'26''E | Quaternary basalts and limestones | *gazella gazella, vulpes vulpes, carnivore indet.,* sheep/goat | 0,708049 (n=11) | 0.000107 | [69] |
