# Supplementary figures and images for: Burying power: New insights into incipient leadership in the Late Pre-Pottery Neolithic from an outstanding burial at Baʻja, southern Jordan
Source: PLoS One. 2019 Aug 28;14(8):e0221171. doi: 10.1371/journal.pone.0221171 (PMC6713438; doi:10.1371/journal.pone.0221171)

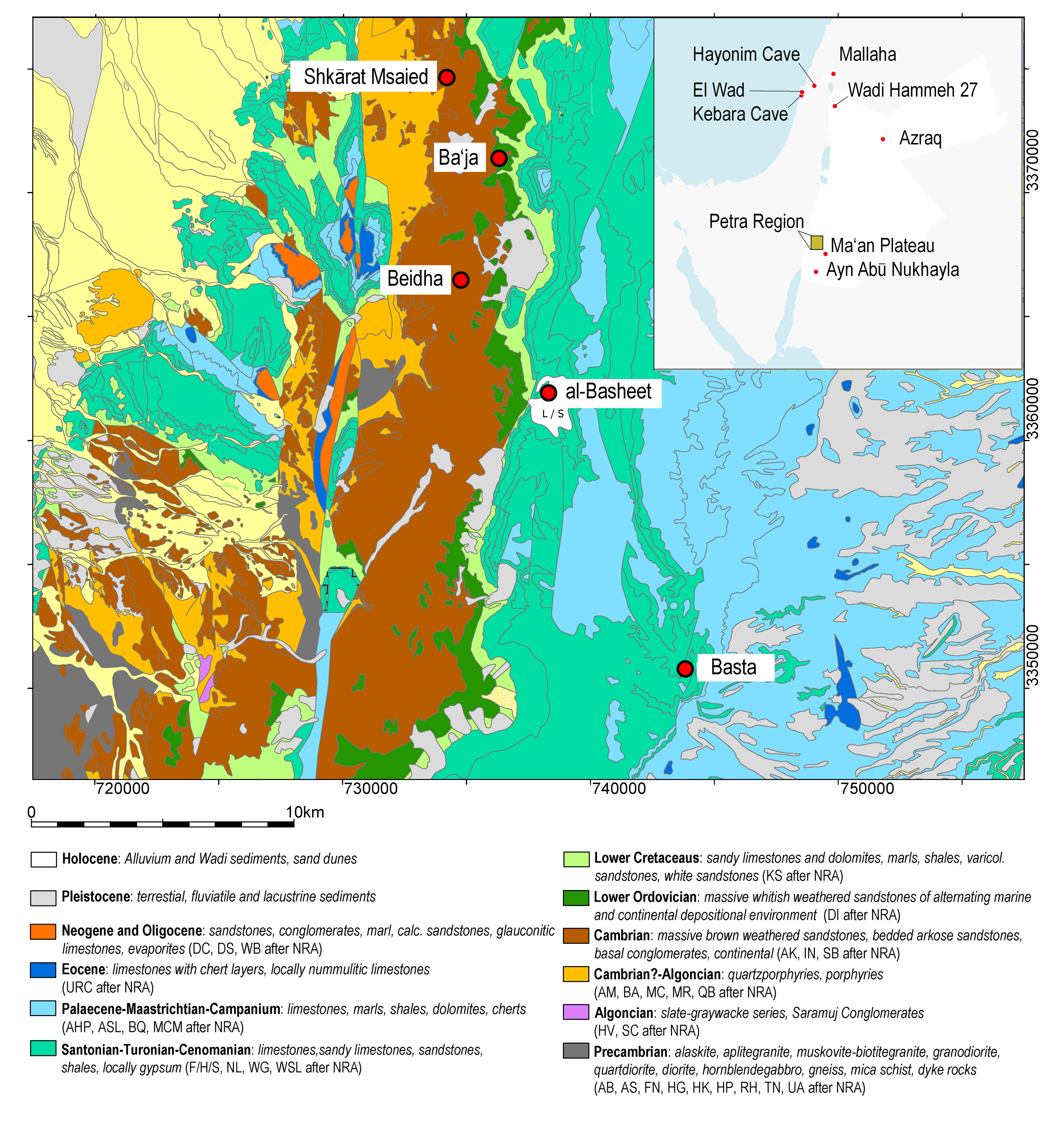

Supplement: S1 Fig — Map designed by C. Purschwitz based on compilation of data from [122–128]; printed under CC BY license, with permission from C. Purschwitz, 2019. (TIF) [file pone.0221171.s003.tif]

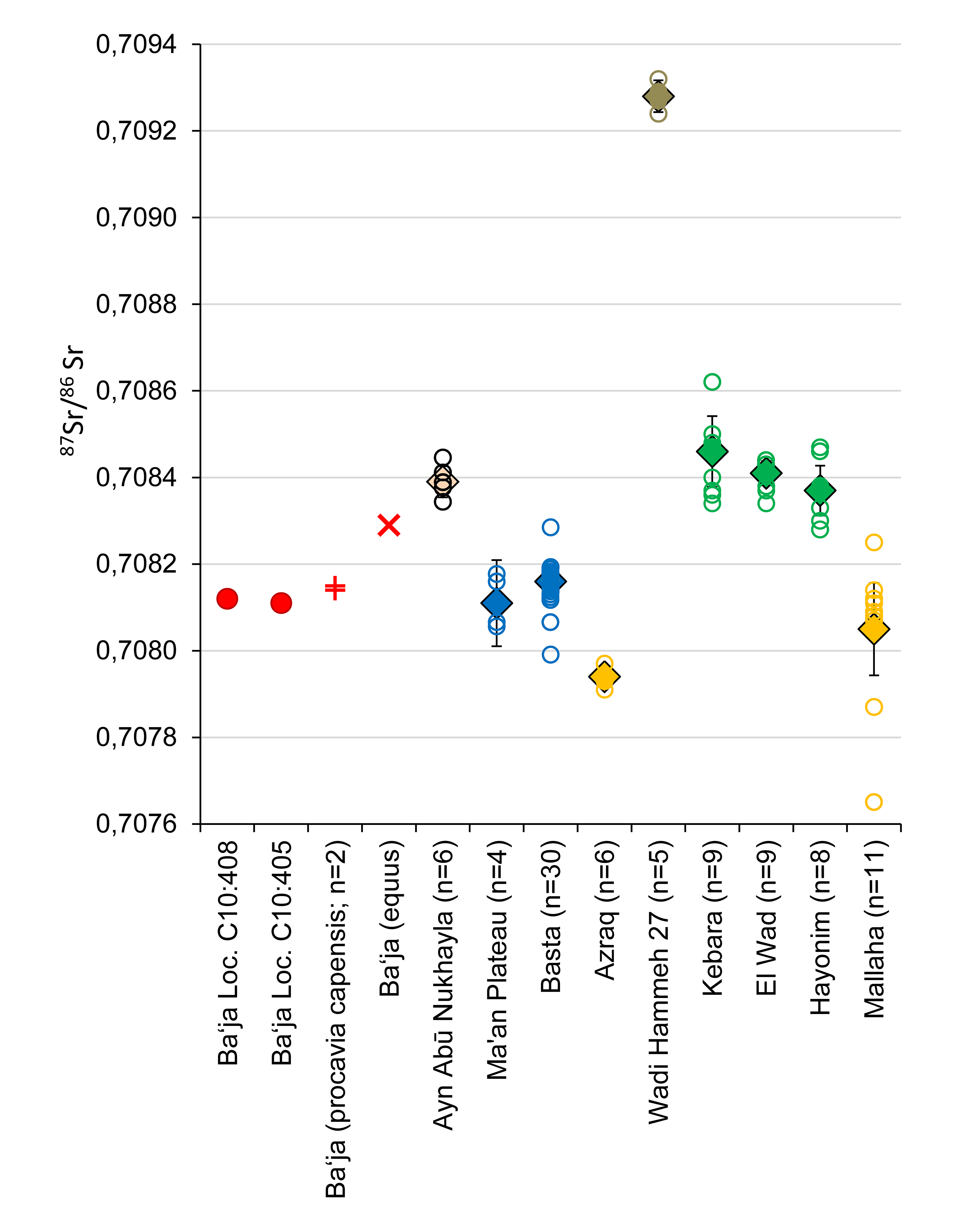

Supplement: S2 Fig — Plain diamonds represent means; error bars represent the 95% confidence intervall for each site. Red: samples from Baʻja analyzed in this text; pinkish: (Pre-)Cambrian sandstones and granitic formations of Wadi Rum; blue/green: limestone formations southeast of Baʻja / in the Mount Carmel Area and Upper Galilee; orange: Quaternary basalts and limestones; brown: travertine deposits. Data sources: [69–70, 129–130]. (Graph: MB/CK). (TIF) [file pone.0221171.s004.tif]

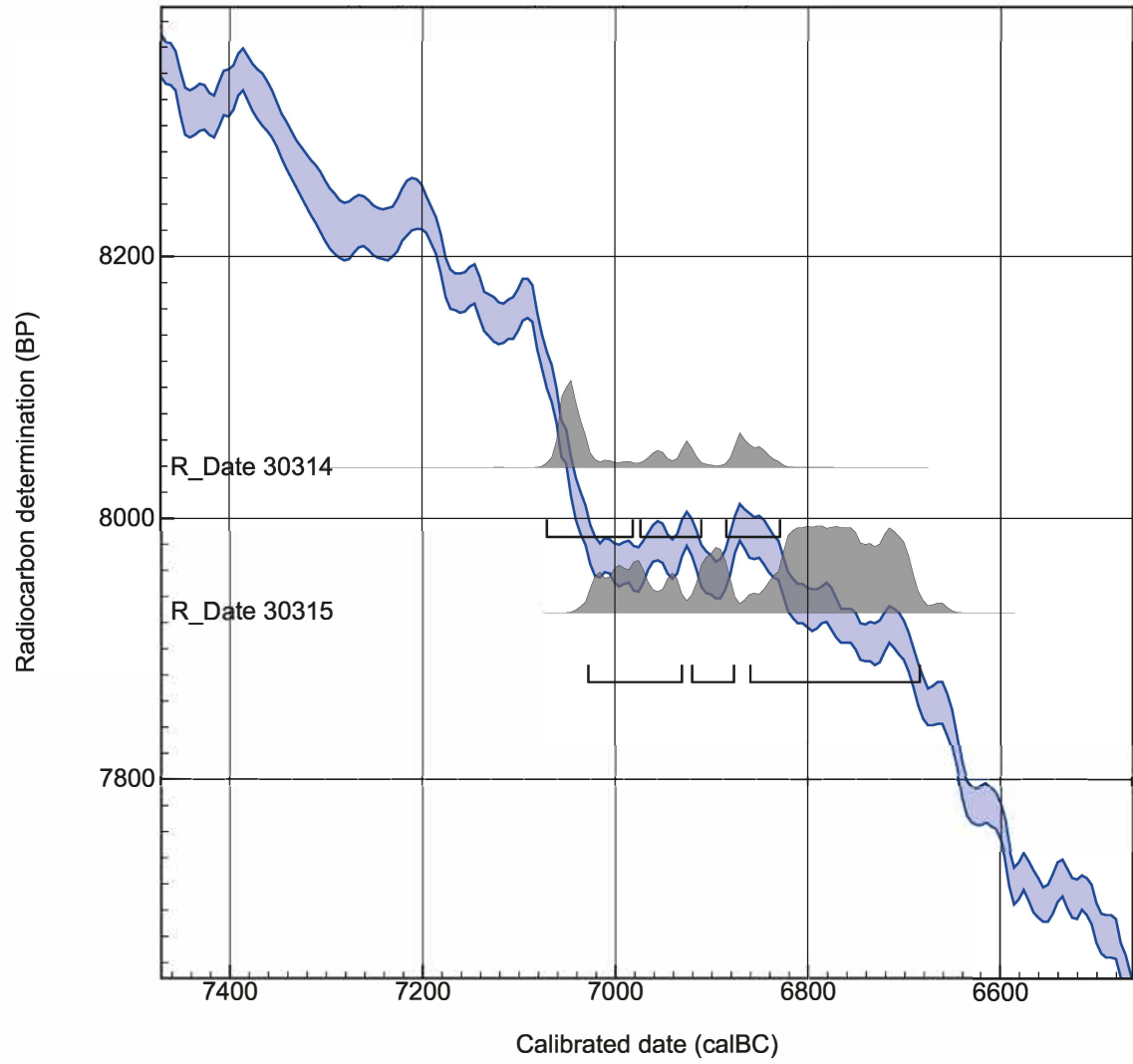

Supplement: S3 Fig — (PDF) [file pone.0221171.s005.pdf]
