# Supplementary material for: Burying power: New insights into incipient leadership in the Late Pre-Pottery Neolithic from an outstanding burial at Baʻja, southern Jordan
Source: PLoS One. 2019 Aug 28;14(8):e0221171. doi: 10.1371/journal.pone.0221171 (PMC6713438; doi:10.1371/journal.pone.0221171)
Supplement: S1 Text — (DOCX) [file pone.0221171.s006.docx]

**S1 Text. Analytical questionnaire for the Burial Loc. C10:408.**

The empirical data of the Burial Loc. C10:408 of Ba´ja were studied according to a standardized catalogue of questions. This study considers the three main categories elaborated in the theoretical discussion (see the *M.A.R.L. Cube*): the leading agency (1=z-axis), pathways to power (2=x-axis) and the socio-political ethos of the community (3=y-axis). For each question values were attributed between -1 and 1 in steps of ±0.25. All values were considered equally. When questions had to be split in several parts, the values were considered as complementary and the median was used for all additional calculations. The medians of all three main categories were calculated. They determine the position of “Baʻja” on the x-, y- and z-axis of the *M.A.R.L. Cube*. The mean absolute deviation from the median is indicated by the error bars in the *M.A.R.L. Cube* (Fig 18).

Even though there are no quantifiable measurements, the qualitative analysis based on the standardized questionnaire aims at a systematic comparison of different case studies. The Burial Loc. C10:408 of Ba´ja should be considered a first case study.

1. **Leading Agency (z-axis)**

**1.1. Material display of power** (scale: -1=very different; +1=not at all different)

- *Space and construction: do spaces occupied by the outstanding person differ from other persons or groups*?

The location of the burial is in the same space but separated from other burials. Despite a lot of variation in burial rituals, burying the dead in between houses or beneath house floors had been a common practice during the PPNB. Isolated cemeteries at the edge of a village seem to develop only during that period [106].
The grave construction differs in elaboration but not in general style from other burial constructions ([19] with further references]); it is almost identical to a recently discovered elaborate single burial of an infant (see Table 5). However, cist grave constructions were exceptional at Baʻja, but they were more common at Shkārat Msaied [46] or El Hemmeh [76].

- *Material culture: do objects used by or offered to the outstanding person differ from common utensils in households or other graves*?

The grave goods comprise objects of high exotic and symbolic value (macehead, green stone, carnelian and shell beads, mother-of-pearl objects, composite arm ring, dagger) with items of daily use (bone spatula, pestle, vase fragment). Highly symbolically laden objects (projectile points and macehead) were deliberately made unusable. The stone vessel fragment and the spatula find good parallels in other objects on the site. Similar categories of objects have been found in other graves (dagger, projectile points, beads, mother-of-pearl items). The use of red pigments is also very common.

The raw material of the arm rings, beads, macehead, pestle and dagger were of non-local provenience. In contrast to the very skilled technology of the dagger, the projectile points are less elaborate than Amuq points, which is the most common type at Baʻja, but this difference might have chronological reasons.

- *Personal adornments: do personal adornments of the outstanding person differ from common adornments on the site or of the culture*?

Greenstone beads are very rare at Baʻja, yet rather common at others sites such as Shkārat Msaied, Beidha and Basta [84-86, 88-90]. They hint at regional and supra-regional networks to which the buried person had access to. Carnelian beads are very rare in the region.

It has to be emphasized that none of the used materials has been exclusive to the outstanding individual, but the style of the composite upper arm ring is unique.

- *Does the burial ritual differ from other burials*?

The individual had been buried according to the local tradition in a crouched body position. However, the single interment sets him apart from almost all other burials which were all collective, except for two other single burials [58-59]. The lack of red coloring of the bones is in contrast to the other burials at Baʻja, but the piece of red mineral between the fingers of the right hand of the individual underlines the importance of pigments in the burial ritual. The separation of two categories of grave goods has been observed only in the lavishly decorated infant burial in the neighboring room (Table 5) and at Kfar haHoresh [80].

The use of fire has also been observed near the double infans I burial, and nearby two collective burials and two recently discovered burials (see Table 5). The burial thus forms a separate, but rather standardized burial type, which abides in many respects to the local burial tradition, even though it segregates the outstanding individual from the group.

For the statistical analyses (Table A) the four questions were subdivided in five domains and the median was calculated: a) style, b) size, c) raw material, d) technology, e) number/effort.

The high mean deviation of the median for all values of the material display of power (=0±0.65) mirrors the high variability in the subcategories.

**Table A.** **The use of space and the display of objects in the burial ritual**.

|  | Style | Size | Material | Technology | Number/  Effort | Median | Mean absolute deviation |
| --- | --- | --- | --- | --- | --- | --- | --- |
| Space | -0.25 | 1 | --- | --- | -1 | -0.25 | 0.75 |
| Architecture |  | | | | | | |
| Grave construction | -0.5 | 0 | 1 | 1 | -0.5 | 0.00 | 0.60 |
| Objects | 1 | 1 | -0.5 | 0 | -0.5 | 0.00 | 0.60 |
| Personal adornments | -1 | -0.5 | 0.5 | 0 | -0.5 | -0.5 | 0.40 |
| Burial ritual | 1 |  |  | 1 | -1 | 1 | 0.67 |
| Median of all subcategories | | | | | | 0 | 0.65 |

Evaluations are based on steps of ±0.25.

**1.2. Anthropological criteria** (-1= yes; +1=no)

- *Physical properties: does the individual differ in physical properties: a) robustness, b) task related traits, c) body size, d) pathologies from the common traits of the group?*
- *Does the person differ in nutrition (C/N isotopes/tooth wear)?*
- *Does the person differ in provenience/mobility patterns?*
- *Familial relations: a-DNA or epigenetic markers*

The poor preservation of the bones hampered valid results from stabile isotope analyses for nutrition (C/N) from bones. The severe wear of the front teeth is in contrast to the rather weak wear of the premolars and molars. The analysis of aDNA from the second upper left molar (27) failed to produce valid results. Thus only the first and third question can be addressed with our data. The diameter of the upper arm rings might point to a gracile adult person, if the rings were worn or put on before decay of body tissue. Whether gracility was a typical trait of the whole community has to be clarified by further anthropological analyses. This trait might have set him apart from a farming community, in which daily workloads required physical strength.

The results of the strontium isotope analyses point to a local individual.

The investigable physical properties (Table B) thus differed only slightly from other Neolithic communities (median=0.38±0.63). Physical properties themselves were thus not primarily decisive, but it can be surmised that the gracility of the individual differed from the rest of a farming population.

**Table B.** **Physical properties and provenience of the outstanding individual compared to other individuals.**

|  | Robust-ness | Task related traits | Body size | Pathologies/  Caries | ^87^SR/  ^86^SR | C/N | Tooth wear | Median | Mean absolute deviation |
| --- | --- | --- | --- | --- | --- | --- | --- | --- | --- |
| Physical properties | -0.25 |  | 1 |  |  |  |  | 0.38 | 0.63 |
| Diet |  |  |  | -0.25 (splitting) |  |  |  | -0.25 | 0 |
| Provenience/  Mobility |  |  |  |  | 1 |  |  | 1 | 0 |
| aDNA | No data available | | | | | | |  |  |
| Median of all anthropological data | | | | | | | | 0.38 | 0.63 |

Evaluations are based on steps of ±0.25.

**1.3. Representation in imagery**

- *Is the individual represented as an outstanding individual or as a primus inter pares*? (-1= aggrandizer; 1=*primus inter pares)*; no data available.

**1.4. Network within the group/with other groups**

- *Could he/she use certain items exclusively, especially of non-local provenience*? (-1=exclusive to him/her; 1=common in the group)

The individual had access to exotic items, but they were not exclusive to him. All raw materials have also been found in other areas of the site = 1

- *Does the individual possess exotic items*? (1=a few; -1=many relative to the group)

The absolute amount of exotic materials and imported items is slightly enhanced compared to collective burials, but given that the objects were attributed to an individual the relative amount is rather elevated = 0

Median: 0.5, mean absolute deviation of the median: ±0.5

**1.5. Accumulation of wealth**

- *Is the outstanding person allowed to accumulating wealth? Or is there some evidence for communal storage of goods*? (-1= individual accumulation/hoarding; 1= communal storage of goods)

No data available; but the display of accumulated wealth was not an important aspect that was represented during the burial ritual. At least from an ideological point of view, the accumulation of objects was obviously not a criterion for excellence.

The median of all three categories that provided data (1.1.-1.2.; 1.4) for the characteristics of the outstanding person is **0.38 ±0.17**. The deviation represents the mean absolute deviation of the median. The individual thus tends to the *primus inter pares* type, but shows also some clear traits of an individualistic aggrandizer. Whereas physical properties and material goods tend to the representation as *primus inter pares*, the reclaimed space, efforts in grave construction and the display of exotic items inside the grave shift the individual towards the aggrandizer type.

1. **Pathways to power (x-axis)**
   1. **Material culture**

Results of the two first questions should be considered as complementary.

- *Do weapons or other items of potential harm play a role a) in imagery, b) in personal adornments*? (-1= very important; 0= no)

a) There are hardly any data on imagery in the archaeological records; b) both categories of grave goods comprise potential weapons: two projectile points, a dagger and a macehead. Except for the dagger, all three other items were destroyed deliberately. Microscopic analyses show that the dagger was not used often, but possibly made for ritual or symbolic purposes. All four items were made of non-local material. It thus seems that these objects were important and possibly made for status or ritual purposes. Whereas the dagger might have had a ritual function, it is more difficult to imagine a ritual function for the macehead. With the death of the person, they were made unusable, i.e. the power they represented should not be transferred to someone else = -0.75.

- *Are mitigating scenes or other themes of detention/feasting displayed a) in imagery , b) personal adornments*? (0=none; 1=very important); a) no data; b) no data.
- *Is there some evidence for psychological indoctrination (e.g. canonization of symbols, monumental art, representation of certain individuals etc.)?* (-1=yes strongly; +1=not at all). There is no evidence of canonization in symbolism indicating doctrinal modes of ideology = 1.
- *Are objects reclaimed exclusively by the individual?* (-1=yes; +1=no). Access to exotic objects was open to other persons too =1.
- *Is accumulation (and display) of economic wealth important*? (-1= of high importance; +1= of no importance). At least after death, the display of accumulated wealth had no meaning. Respect and commitment was displayed by the efforts put in the grave construction =1.
- *Are objects transferred to the next generation or are they ex-commodified*? Many objects and even whole households seem to be ex-commodified (*sensu* [26]) =1.

In general, the material remains do not speak in favor of *coercion* for the pathway to power, except for the potential weapons which were associated with the individual, but which were destroyed deliberately (median=**1±0.35**) (Table C).

**Table C.** **Material evidence for the pathway to power.**

| Evidence for pathways to power in the material records | Estimated value | Mean absolute  deviation |
| --- | --- | --- |
| Display of weapons/mitigation | -0.75 |  |
| Indoctrination *vs.* broad spectrum of symbolism | 1 |  |
| Restricted access *vs.* free access | 1 |  |
| Costly display *vs*. “understatement” | 1 |  |
| Transfer of goods over generations *vs.*  ex-commodification | 1 |  |
| Median | 1 | 0.35 |

Evaluations are based on steps of ±0.25. Note that the mean deviation of the median in the *SPIEL-Cube Model* cannot exceed 1.

**2.2. Anthropological criteria**

- *Do individuals especially juveniles and adults show signs of mutilations or injuries due to physical force*? No data.
- *Is biological relationship decisive for status*? No data.
- *Is bodily excellence a criterion*? As with many other early Neolithic individuals, the teeth of the individual show some hypoplasia during childhood indicating stresses in nutrition or because of diseases. Other signs of malnutrition could not be observed due to the poor preservation. The suggested gracility (see above) might indicate that physical strength was not a decisive factor.
- *Did pathologies play a role*? No serious pathologies have yet been detected (possibly hindered by the poor preservation of the skeleton), which meant a visible handicap or influenced the bodily functions of the individual. However, it should be emphasized that many diseases, including many neurobiological dysfunctions, leave no traces in the anthropological records.

The only slight evidences from the anthropological data is, that physical strength obviously was not a decisive criterion, however due to the poor preservation of the bone surfaces and the lack of secure comparisons with other individuals from the site, this criterion has not been considered for the calculation. Further analyses of aDNA would be of high priority for gaining more information on that point.

1. **Socio-political ethos (y-axis)**
   1. **Material culture**

- *Were there strong conventions/traditions* in: a) *architecture, b) objects, c) imagery, d) settlement layout?* (-1 =none; 1=very homogenous)

1. The architecture of the site manifests certain basic rules and technological skills with probably preplanned structures, but there are also a lot of “bricolage”/ad hoc constructions of various styles. Space had been constantly reshaped by adding new walls, closing of windows, etc. and functions of space had been changed repeatedly. The coherence and overall layout of the village seems to form a rather homogeneous whole =+0.5.

With few exceptions no communal architecture has yet been detected =-0.75.

1. Objects seem to be distributed rather evenly over the whole site. There seems to be no single household that is set apart by different traditions. On the contrary, important objects such as the sand stone rings were produced and discarded everywhere on the side in domestic contexts. Some households seem to have had access to better flint resources, but there was no exclusive use visible. A few objects were very standardized (e.g. mother-of-pearl amulets, daggers), but there was also an ad hoc, rather individualistic production of objects for domestic use following certain technological traditions but without a strict canonization =0.

Some of the grinding stones are so huge, that they might suggest communal processing of food, but the evidence remains poor.

1. no data
2. the general style of the settlement resembles other Late PPNB settlements, but there is so much individualistic planning that the inner structure of the settlement differs considerably from other settlements such as Basta, ‘Ain Jammam, Beidha or Ghweir in southern Jordan =0.

- *Were boundaries displayed in a) architecture (within the settlement), b) settlement layout (corporate identities), c) imagery?* (for a: individual boundaries: -1=yes, 1=no; for b: collective boundaries: -1 =no, 1=yes)

1. Room entities were separated, but they were so close to each other that there was no space in between. Access was probably through roofs and through so called window openings. Roofs played an important role in communication, but it is unclear, whether roof space was communal space or individualized per social entity =0.5.
2. The siq formed a steep natural boundary and the agglutinating architecture made the village appear like a fortress. There were only a few openings enhancing the impression of a rather closed entity =1.
3. Imagery: no data available.

NB: values on the scale of a) and b) must be opposed. If there had been intra-site boundaries, individual/familial properties would have been segregated; if there had been communal boundaries, the whole community would have been manifested as a coherent entity or protected.

- *Accumulation of wealth:*

*Is there communal or private storage of goods? (-1= individual accumulation/private hoarding 1= communal storage of goods);*The hoarding of objects was observed in several instances but no supra-household collective storeroom has yet been found. If there was hoarding or depositions of accumulated objects, it seems to have been on the household level. Most of the individuals were buried in collective burials depersonalizing all personal property, if it had existed before =0.25.

NB: This point is no option for mobile hunter-gatherers and therefore has to be neglected in the study of small scale mobile foraging people.

- 1. **Anthropological criteria:**
- *Are populations homogenous or heterogeneous:
  a) in a-DNA, b) nutrition (C/N), c) mobility patterns, d) epigenetic traits/physical appearance*? *(-1= very heterogeneous; +1= very homogeneous*); no data are yet available.

The median for the available data of category 3 (Table D) tends to a collective ethos of the community (median=**0.25±0.25**) but decisive data, especially from the anthropological records, are still missing to confirm this trend.

**Table D. Collectivism *vs*. individualism expressed in the settlement and other material remains.**

| Material goods | Standardization (1) *vs.* individualistic (-1) | Collective (1) *vs*. individual (-1) | Median | Mean  absolute deviation |
| --- | --- | --- | --- | --- |
| Architecture (a) | 0.5 | -0.75 | -0.13 | 0.63 |
| Objects (b) | 0 | ---- | 0 | 0.00 |
| Imagery (c) | ---- | ---- |  |  |
| Settlement (d) | 0 |  | 0 | 0.00 |
| All material goods | | | **0** | 0.42 |
| Display of boundaries | Collective (1) *vs.* individualistic (-1) | | | |
| Architecture (a) | 0.5 | | 0.5 | 0 |
| Settlement (b) | 1 | | 1 | 0 |
| Imagery (c) | ---- | |  |  |
| Display of boundaries in all investigated categories | | | **0.75** | 0.25 |
| Accumulation of wealth | Collective (1) *vs.* private (-1) | |  |  |
| Storage |  | | **0.25** | 0 |
| Anthropological data | Meta-analyses are missing for the LPPNB | | | |
| All subcategories |  | | **0.25** | **0.25** |

Evaluations are based on steps of ±0.25. Imagery and anthropological data were not considered due to the rarity of data and missing meta-analyses.
