# Supplementary material for: Burying power: New insights into incipient leadership in the Late Pre-Pottery Neolithic from an outstanding burial at Baʻja, southern Jordan
Source: PLoS One. 2019 Aug 28;14(8):e0221171. doi: 10.1371/journal.pone.0221171 (PMC6713438; doi:10.1371/journal.pone.0221171)
Supplement: S1 Protocol — (DOCX) [file pone.0221171.s007.docx]

**S1 Protocol. Sample preparation for strontium isotope analyses.**

Sample preparation followed the methods described in Knipper et al. [1-2] and comprised the following steps:

Enamel fragments were cut from the tooth crowns using a diamond-coated cutting disc attached to a dental drill and all surfaces and remaining dentin were removed using diamond-coated milling bits. The samples were ground in an agate mortar. Eleven to 12 mg of sample material were weighed into sample tubes and pre-treated in successive steps with 1.8 ml of Milli-Q water, 1.8 ml of 0.1 M acetic acid buffered with lithium acetate (pH ca. 4.5) and washed three times with 1.8 ml of H_2_O. During each of these steps the samples were placed into an ultrasonic bath for 10 min. The objective of the pre-treatment procedure was to remove diagenetic carbonates. Samples were dried afterwards overnight (50°C) and ashed to remove remaining organic components (3 h at 850°C).

All subsequent steps were carried out under clean lab conditions. The samples were dissolved in nitric acid (3 N HNO_3_) and the strontium separated using Sr-Spec ion exchange resin. Strontium concentrations were determined using a Quadrupol ICP mass spectrometer, the solutions diluted, and the isotope ratios determined using a High Resolution-Multi Collector-Inductively Coupled Plasma-Mass Spectrometer (HR-MC-ICP-MS; Neptune). The raw data were corrected according to the exponential mass fractionation law to ^88^Sr/^86^Sr = 8.375209. Blank values were less than 10 pg Sr during the whole clean lab procedure, including digestion, Sr separation and measurement. Standards run with the samples produced the values listed in Table A.

**Table A.** **^87^Sr/^86^Sr ratios of the NBS 987 and Eimer and Amend standards run with the samples***.*

| Standard | Number | ^87^Sr/^86^Sr Avg | 2 Sigma |
| --- | --- | --- | --- |
| AMES | 3 | 0.70805 | 0.00001 |
| NBS-987 | 4 | 0.71027 | 0.00001 |

**References**

1. Knipper C, Maurer A-F, Peters D, Meyer C, Brauns M, Galer SG, et al. Mobility in Thuringia or mobile Thuringians: a strontium isotope study from early medieval central Germany. In: Kaiser E, Burger J, Schier W. editor. Migrations in Prehistory and Early History. Stable Isotopes and Population Genetics. Berlin: De Gruyter; 2012. pp. 287-310.
2. Knipper C, Meyer C, Jacobi F, Roth C, Fecher M, Schatz K, et al. Social differentiation and land use at an Early Iron Age "princely seat": bioarchaeological investigations at the Glauberg (Germany). J Archaeol Sci 2014;41: 818-835.
